# Toxicological Evaluations Following 28-Day Oral Gavage Exposure to Polyethylene Terephthalate Microplastic Particles in Mice

**DOI:** 10.3390/ijms27219397

**Published:** 2026-10-22

**Authors:** Kristen A. Marcellus, Michal Scur, Shahad Abdulmawjood, Gen-Sheng Wang, David Prescott, Jith Thomas, Nikia Ross, Santokh S. Gill

**Affiliations:** Food Safety Regulatory Research Division, Bureau of Microbial and Toxicological Food Safety, Health Products and Food Branch, Health Canada, Ottawa, ON K1A 0K9, Canada

**Keywords:** microplastic, nanoplastic, polyethylene terephthalate, systemic toxicity

## Abstract

The degradation of plastic in the environment can result in the generation of micro- and nano-plastics. As one of the common types of plastics used, polyethylene terephthalate (PET) microplastics are pervasive in the environment. Yet, their potential toxicity remains poorly understood. In this study, we addressed this gap by examining systemic toxicity in C57BL/6 mice following 28-day oral gavage with irregularly shaped PET microplastics at human-relevant exposure levels of 0, 0.3, 1.5, 7.5, or 37.5 mg/kg bodyweight/day. No significant changes were seen in bodyweights, food consumption, organ weights, clinical biochemistry, or hematological parameters. Histological changes were seen in the kidneys of male mice with minimum to mild apoptosis in the outer strip of the outer medulla. Additionally, decreased goblet cells were observed in both male and female mice, albeit in different intestinal sections. However, this change in goblet cell population did not result in overt epithelial damage. Overall, sub-chronic PET microplastic exposure did not cause overt systemic toxicity, but sex-specific minor histological alterations in the kidney and gut were observed. Our findings show only minor effects following short-term exposure, but real-world microplastic exposure is likely to be chronic, thus a longer exposure period may be necessary to fully elucidate the potential impact of PET microplastic-induced toxicity.

## 1. Introduction

Microplastics (MPs) and nanoplastics (NPs) are plastic particles with diameters of <5 mm and <100 nm, respectively [1,2,3]. These plastic particles arise from either commercial production for industrial use, termed primary MPs, or secondary MPs from the degradation of larger plastic items by environmental conditions, such as photolysis, thermo-oxidative degradation, biodegradation, mechanical fragmentation, and hydrolysis [4,5,6,7,8,9]. Micro- and nano-plastics (MNPLs) have been found in seawater, freshwater, soil, and air, as well as in remote regions [3,4,8,10,11]. As a result of the ubiquitous presence of MNPLs in the environment and the reported presence of MNPLs in various food products, human exposure may occur via ingestion and inhalation [5,7,12,13]. In fact, MNPLs have been reported in human stool samples, bodily fluids, and various other tissues [14,15,16,17,18,19,20,21,22,23,24,25,26,27,28,29,30,31,32]. Nevertheless, the potential impact of MNPLs on human health remains unknown.

Upon ingestion, a small fraction of MNPLs < 150 µm are thought to be able to translocate through the gut epithelium and enter circulation, and MNPLs < 1.5 µm have the potential to accumulate in organs, while the rest of the particles (>90%) are excreted [1,13,33,34,35]. Recent studies using in vivo mammalian models have shown that MNPL particles accumulate in the heart, liver, kidney, lungs, brain, stomach, gut, spleen, and reproductive organs [3,36,37,38,39,40,41,42]. Once these MNPLs penetrate tissues, they have the potential to cause physical and mechanical damage and can induce oxidative stress, gut dysbiosis, intestinal barrier dysfunction, neurotoxicity, liver metabolic disorder, kidney injury, and inflammation, disrupt energy homeostasis, and alter gene expression [11,33,35,40,43,44,45,46,47]. This highlights the need for data on absorption and further research on the potential toxicological effect of ingested MNPLs on human health.

The toxicity of MNPLs is dependent on their physical and chemical properties [47,48,49]. These properties encompass polymer composition, size, and shape, as well as surface properties of the MPs/NPs [33,47]. Secondary MNPLs develop diverse shapes and surface characteristics after undergoing environmental weathering processes [50]. Particularly, size (nano and micro) and shape may impact particle–biological system interactions, biodistribution, and toxicity [3,13,34,51,52]. In addition, toxicological information about “environmentally weathered” MPs/NPs is poorly documented. The lack of standardization of what “weathering” entails and the complexities of generating these plastic particles have contributed to underutilization of these types of particles in rodent studies. Commercially available particles, uniform in size and shape with smooth surfaces, do not accurately reflect particles humans may be exposed to.

As a widely used plastic in consumer goods and packaging, polyethylene terephthalate (PET) is one of the most commonly found MPs in the environment [9,53,54,55]. Despite this, the subacute hazards associated with PET-MP exposure in vivo are not well characterized, particularly for irregularly shaped microparticles, which are known to have different toxicological characteristics from their spherical counterparts. To remedy this, we investigated the toxicological effects of irregular PET particles in an OECD TG407-based repeated-dose 28-day oral toxicity study employing 4 doses of particles (0.3, 1.5, 7.5, and 37.5 mg/kg bw/day) that were chosen based on current estimates of human exposure levels [55,56,57]. This study is among the first to provide a comprehensive, guideline-directed assessment of PET toxicity by analyzing organ coefficients, hematological and biochemical parameters, immune cell populations, and organ histology.

## 2. Results

### 2.1. Characterization of PET-MPs

**Figure 1:**
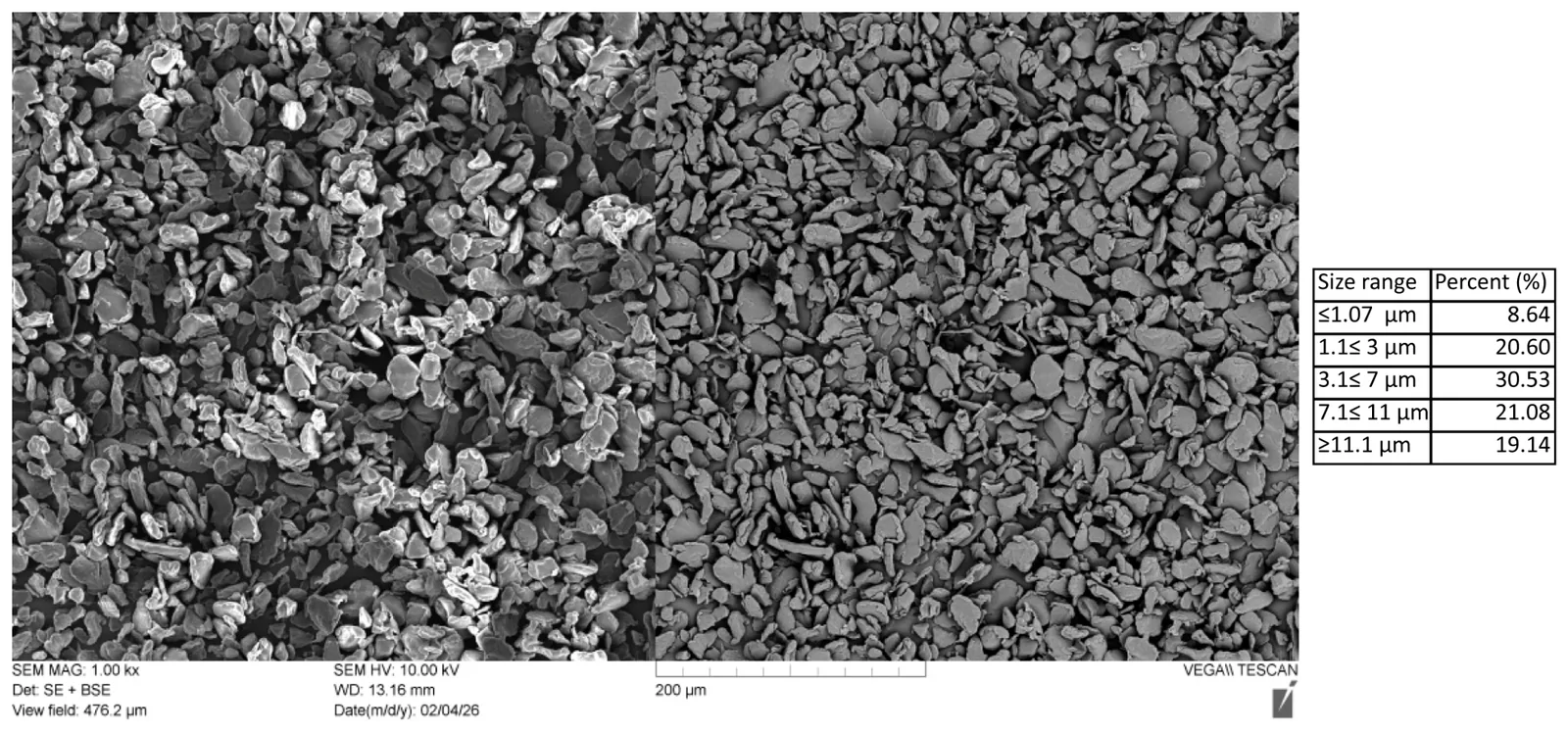
Characterization of PET-MPs. SEM image of PET-MPs and size chart. Left image has higher beam intensity and right image has lower beam intensity.

Scanning electron microscopy (SEM) was used to observe the size and shape of the plastic PET-MPs. Figure 1 displays an SEM image of the plastic particles. The plastic particles were of irregular size and amorphous shape. Image J analysis revealed that overall, approximately 80.1% of the particles were less than 11 µm in size and 29.2% were less than 3 µm. The PET mixture was too heterogenic for dispersion analysis, but zeta-potential was measured using dynamic light scattering. The zeta-potential was −35.67 ± 5.98 mV. PET particles were free of endotoxin contamination.

### 2.2. Bodyweight, Organ Weights, and Food Consumption

**Figure 2:**
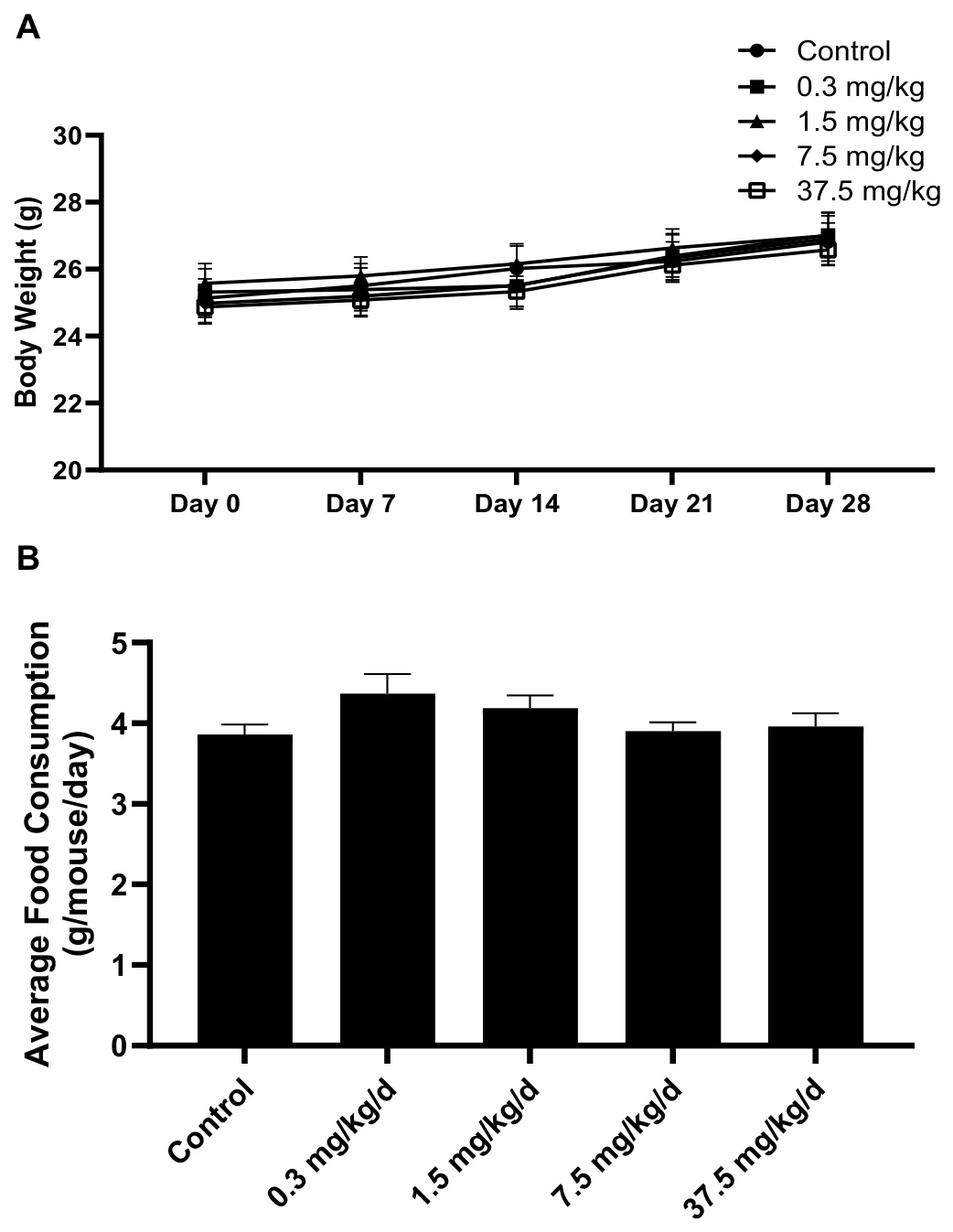
Bodyweights and food consumption of male mice exposed to PET. (**A**) Growth curve of mice weighed weekly over the course of the experiment. Data are the mean ± SEM for *n* = 10 mice/group. Repeated-measures two-way ANOVA suggested that significant effects on bodyweight were observed for time (*p* = 0.0037), but not for dose (*p* = 0.4490) or the time × dose interaction (*p* > 0.9999). (**B**) Average daily food consumption over the course of the experiment, measured weekly. Data are the mean per mouse ± SEM for *n* = 10 mice/group. One-way ANOVA followed by Dunnett’s multiple comparisons test, *p* > 0.05.

**Figure 3:**
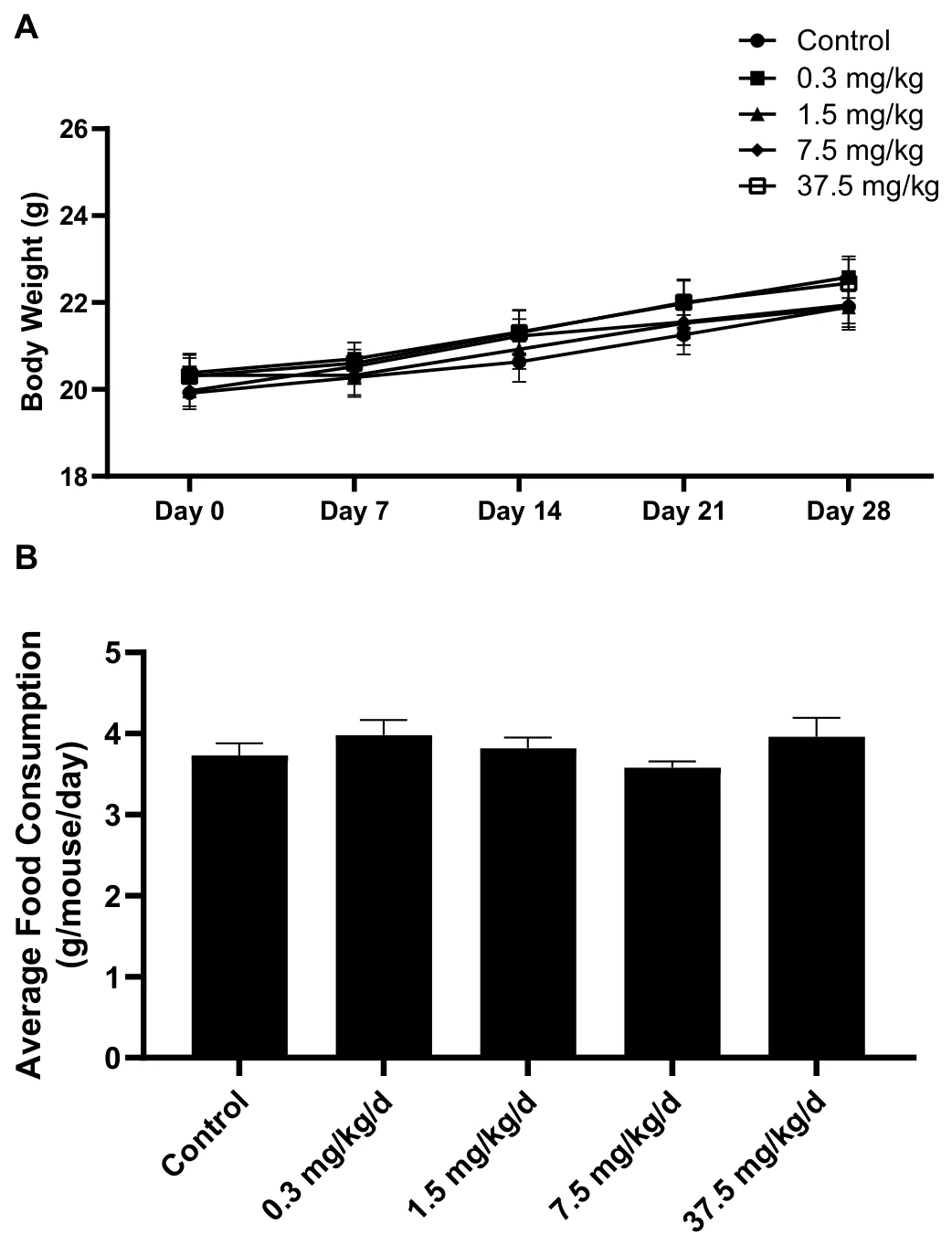
Bodyweights and food consumption of female mice exposed to PET. (**A**) Growth curve of mice weighed weekly over the course of the experiment. Data are the mean ± SEM for *n* = 10 mice/group. Repeated-measures two-way ANOVA suggested that significant effects on bodyweight were observed for time (*p* < 0.0001), but not for dose (*p* = 0.8577) or the time × dose interaction (*p* > 0.8511). (**B**) Average daily food consumption over the course of the experiment. Data are the mean per mouse ± SEM for *n* = 5 co-housed pairs/group. One-way ANOVA followed by Dunnett’s multiple comparisons test, *p* > 0.05.

For the duration of the study, all animals appeared healthy. No change in bodyweight was observed between controls and treatment groups for either sex (Figure 2A and Figure 3A). Food consumption did not differ between the groups (Figure 2B and Figure 3B). No change in organ weight coefficients was noted in either males or females (Table 1 and Table 2). Absolute weight of all organs can be found in the Appendix (Table A2 and Table A3).

### 2.3. Clinical Biochemistry

In order to observe any changes in biological functions due to PET exposure, serum biochemistry was analyzed. No changes in serum clinical parameters were detected in either males or females (Table 3 and Table 4).

### 2.4. Hematology

Hematological tests were performed after PET particle exposure. Minor changes in the neutrophil counts of male mice were observed at 0.3 mg/kg. Total white blood cells and lymphocyte counts were significantly increased in female mice at 37.5 mg/kg. Overall, there were no major changes in hematological parameters in either males or females exposed to PET (Table 5 and Table 6).

### 2.5. Immunophenotyping, Cytokine/Chemokine Expression, and Corticosterone Levels

**Figure 4:**
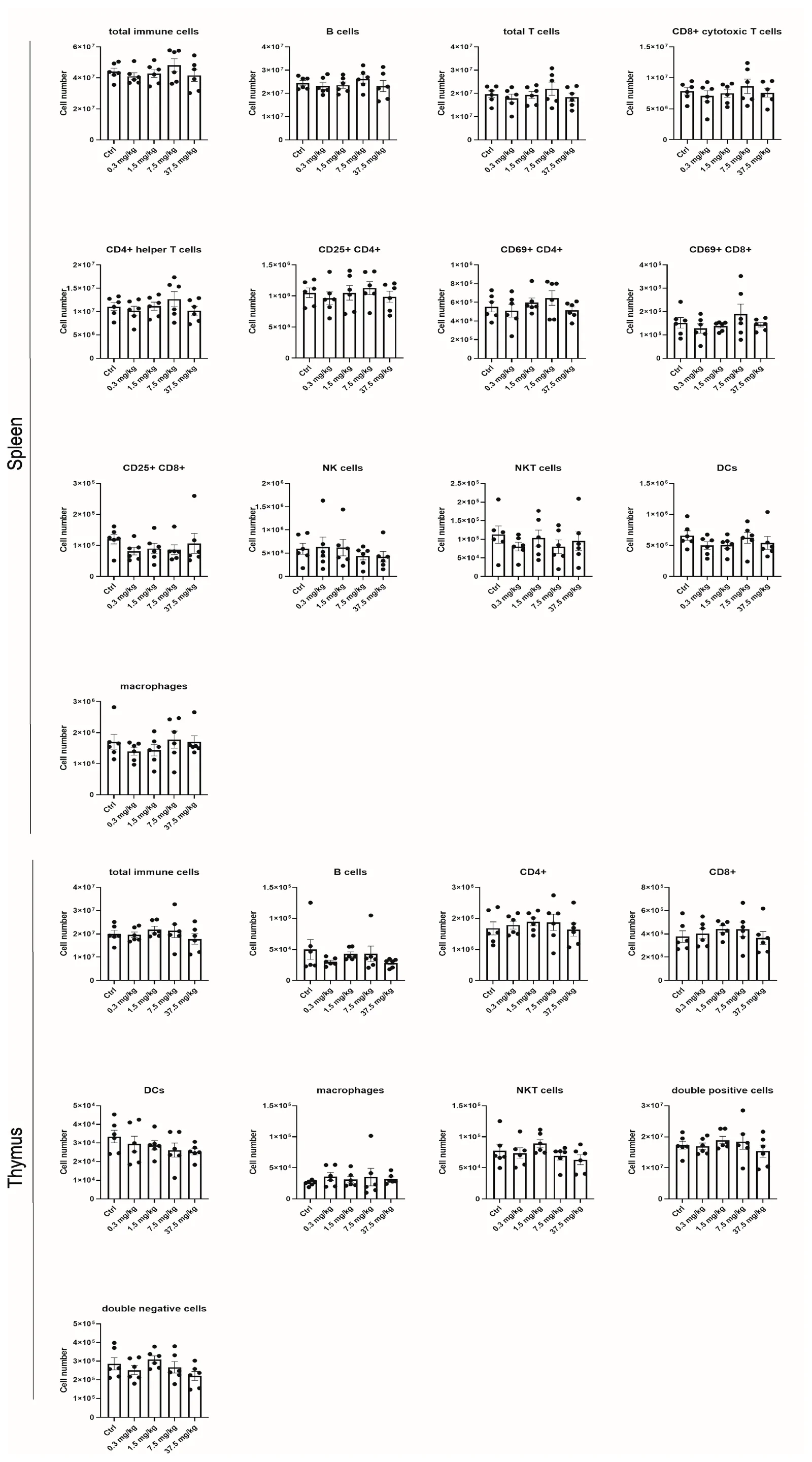
Immunophenotyping of spleen and thymus immune cell populations in male mice exposed to PET for 28 days. Filled circles represent individual mice. Data are the average cell number ± SEM for *n* = 6/group. One-way ANOVA with Dunnett’s post hoc test, *p* > 0.05, not significant.

**Figure 5:**
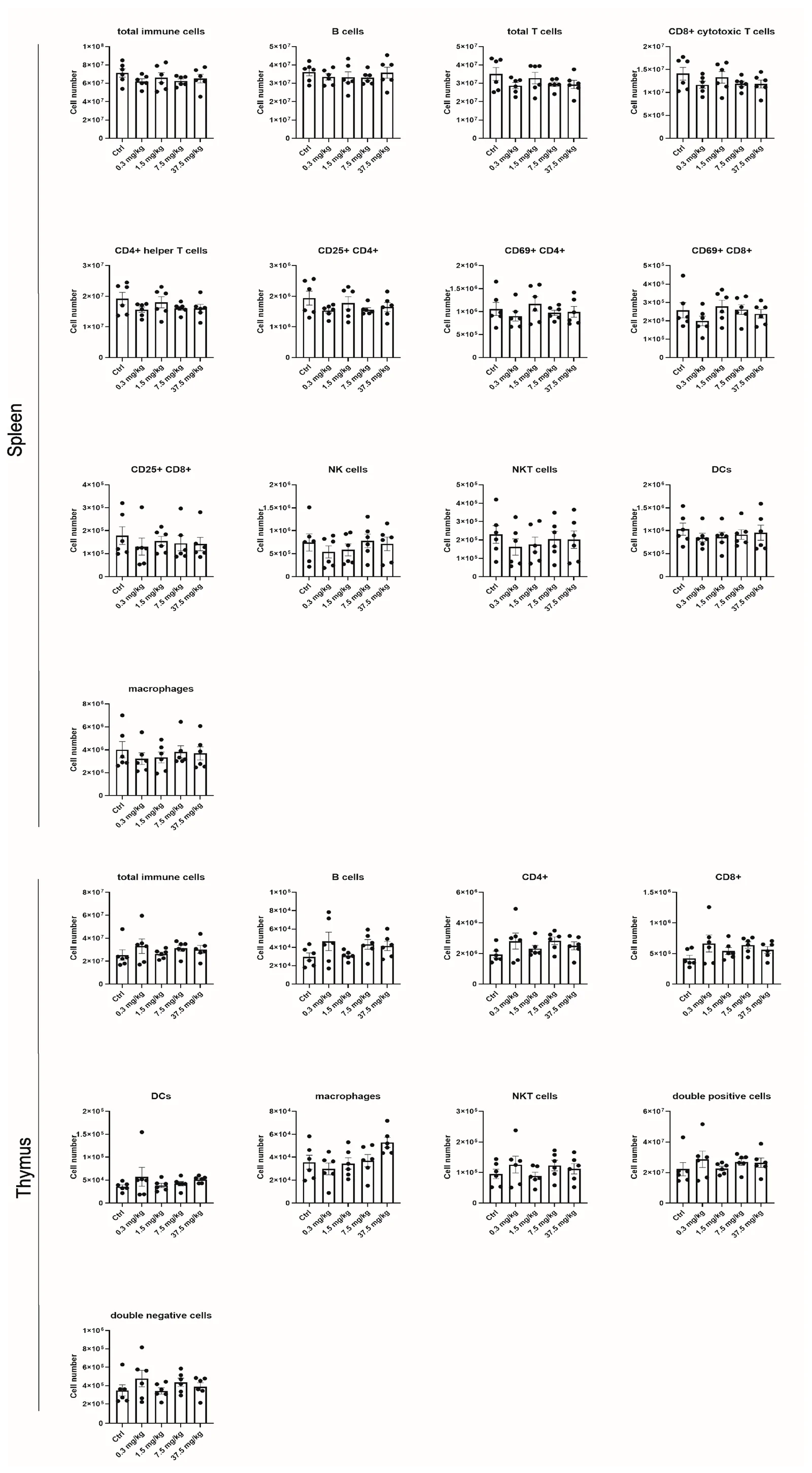
Immunophenotyping of spleen and thymus immune cell populations in female mice exposed to PET for 28 days. Filled circles represent individual mice. Data are the average cell number ± SEM for *n* = 6/group. One-way ANOVA with Dunnett’s post hoc test, *p* > 0.05, not significant.

**Figure 6:**
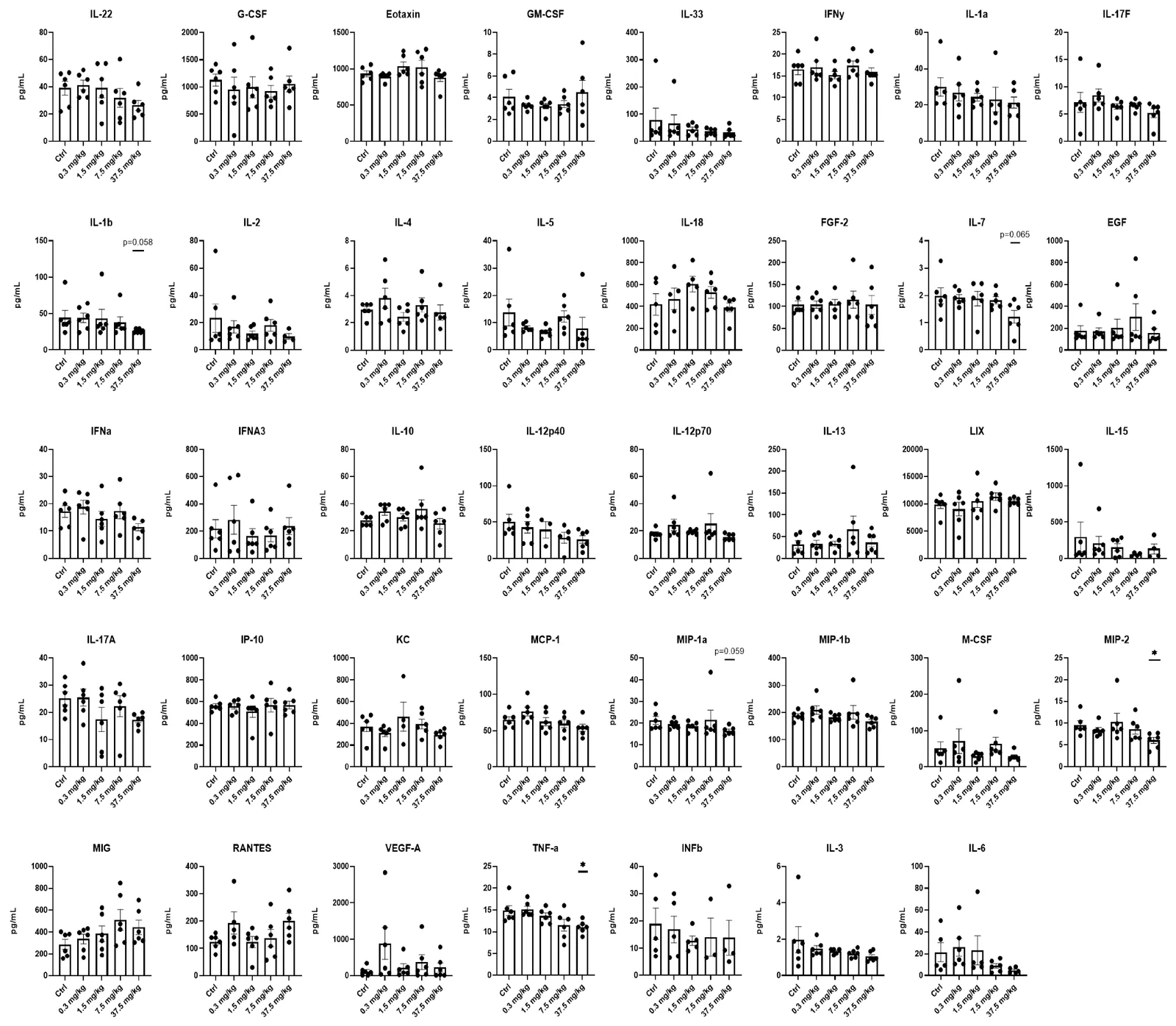
Cytokine and chemokine levels in serum of male mice after 28 days of exposure to PET. Data are the mean ± SEM for *n* = 6 mice/group, except for IL-1α, IL-2, INFβ, IL-4, IL-18, IL-6, FGF-2, INFα, IL-12p40, IL-13, IL-15, KC, and RANTES, which were sometimes below the detection level in an animal. Filled circles represent individual mice. Data were analyzed by one-way ANOVA with Dunnett’s post hoc test, where * = *p* < 0.05. To account for multiple Dunnett’s tests across 39 cytokines and chemokines, the Benjamini–Hochberg procedure for false discovery rate (FDR) correction was performed. No analytes remained statistically significant when the FDR limit was set to 10% (q < 0.1).

**Figure 7:**
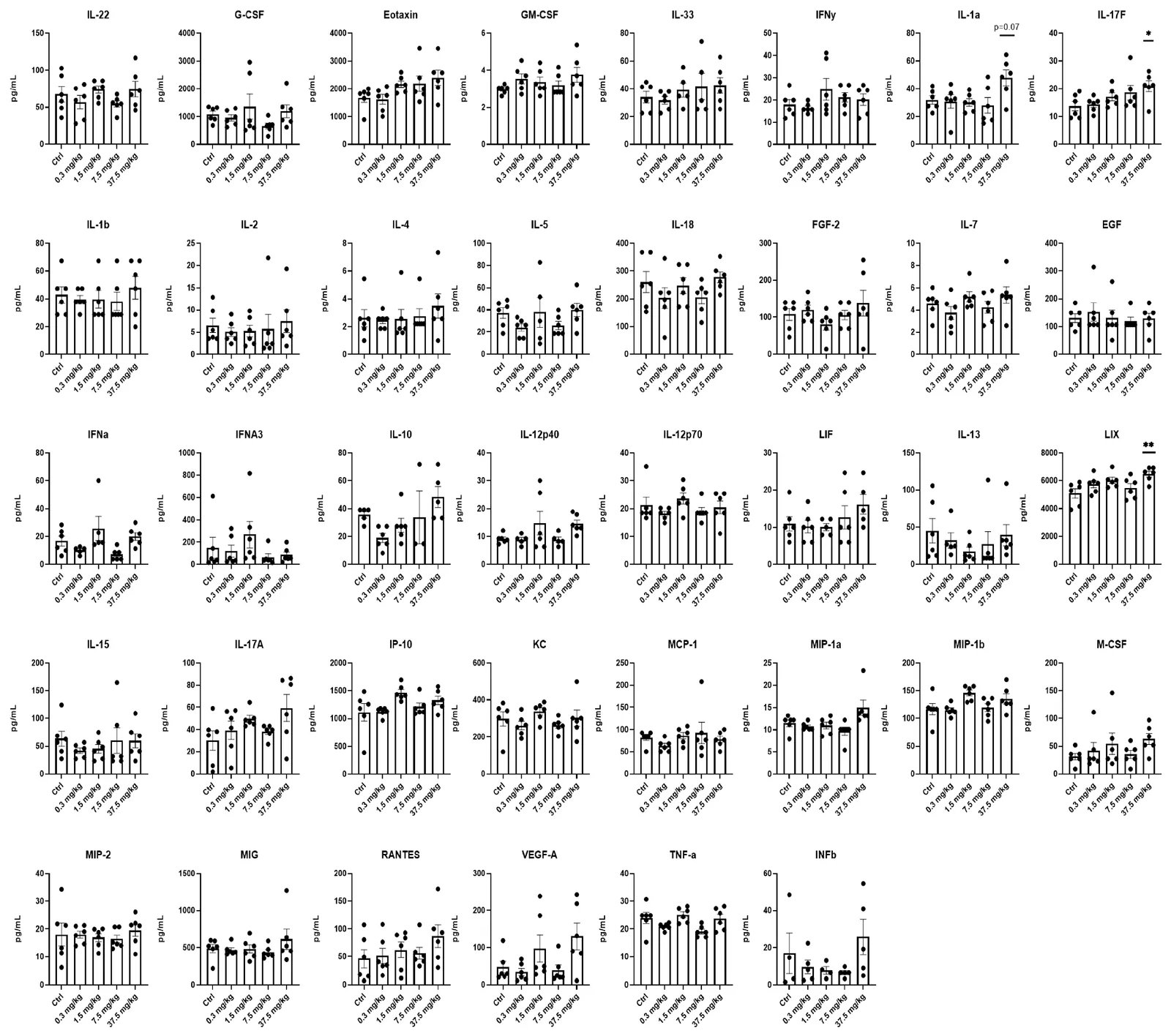
Cytokine and chemokine levels in serum of female mice after 28 days of exposure to PET. Data are the mean ± SEM for *n* = 6 mice/group, except for IL-33, IL-5, INFβ, INFα, IL-10, IL-12p40, LIF, and IL-13, which were sometimes below the detection level in an animal. Filled circles represent individual mice. Data were analyzed by one-way ANOVA with Dunnett’s post hoc test, where * = *p* < 0.05. To account for multiple Dunnett’s tests across 38 cytokines and chemokines, the Benjamini–Hochberg procedure for false discovery rate (FDR) correction was performed. No analytes remained statistically significant when the FDR limit was set to 10% (q < 0.1).

Upon examination of immune cell populations in the spleen and thymus, no significant changes were observed in either male or female mice exposed to PET particles (Figure 4 and Figure 5). Systemic inflammatory markers were measured by analyzing cytokine and chemokine expression in the serum of male and female mice exposed to PET-MPs for 28 days. A 41-plex cytokine/chemokine panel was used to measure these inflammatory markers. Male mice treated with the highest dose of PET showed slight statistical decreases in serum levels of two pro-inflammatory cytokines/chemokines, MIP-2 and TNF-α, when markers were assessed individually by Dunnett’s test. However, these differences were not robust enough to exceed an allowable FDR of 10% when the full panel was corrected for false discoveries by the Benjamini–Hochberg method (Figure 6). A decreasing trend in IL-1β, IL-6, IL-7, IL-12p40, MIP-1α, MIP-2, and TNF-α was also observed when animals were compared across doses in PET-exposed male mice (Table 7). Conversely, female mice showed a significant increase in two pro-inflammatory cytokines, IL-17F and LIX (Figure 7), at the highest dose when markers were assessed individually. These too were not robust enough to exceed an allowable FDR of 10%. Female mice also displayed a trend toward dose-dependent production of Eotaxin, IL-1α, IL-17F, and LIX (Table 8). Out of the 41 markers, 39 were in the detectable range for males and 38 for females, with most having had no significant change.

**Table 8:** Dose-dependent trends in female serum cytokines after PET-MP exposure.

	**Females**	
**Cytokine**	**r**	***p*** ** -Value**
Eotaxin	0.4983	0.0051
IL-1α	0.538	0.0022
IL-17F	0.5039	0.0045
LIX	0.453	0.0119

**Figure 8:**
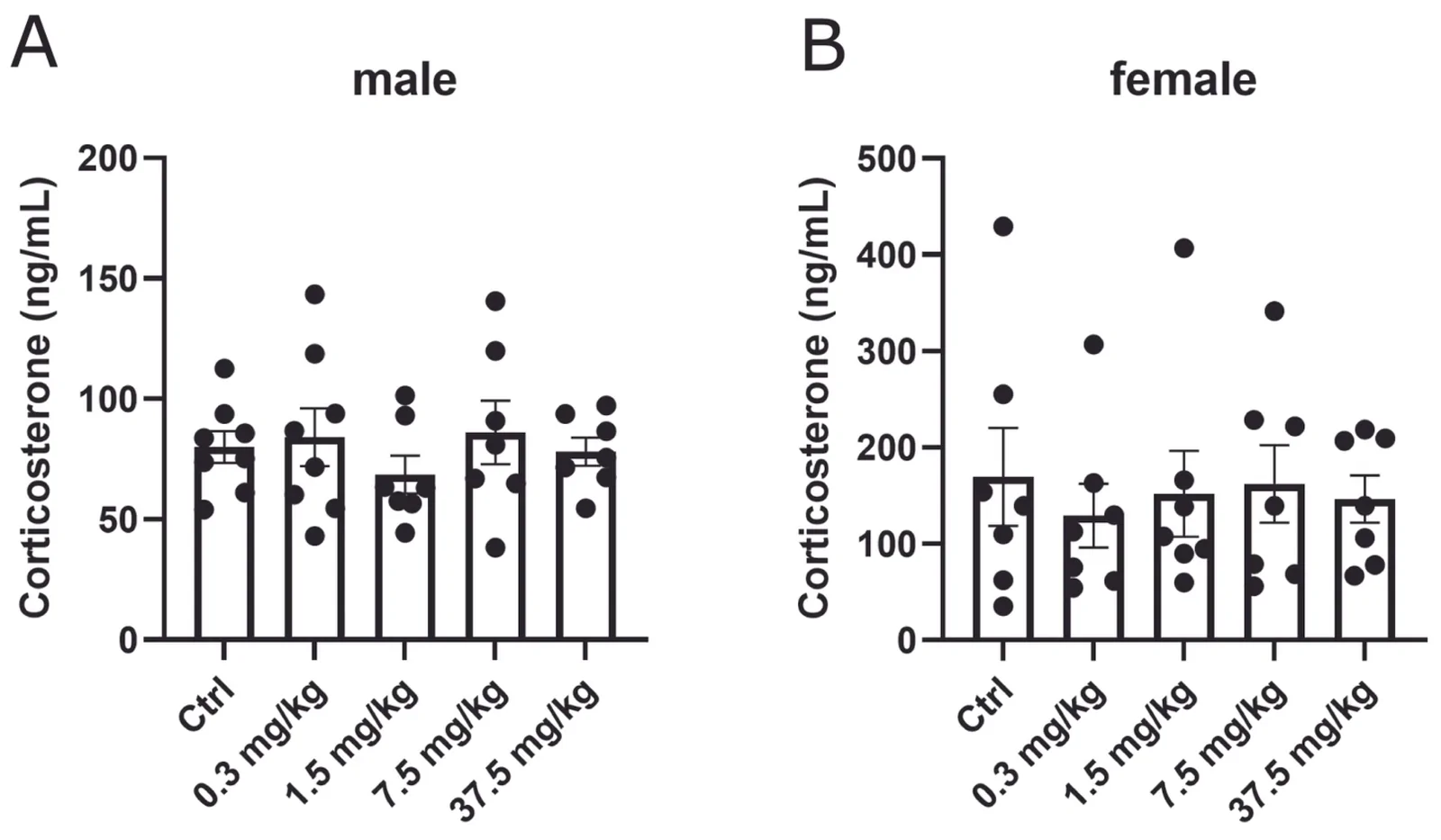
PET exposure does not change corticosterone levels. (**A**) Serum corticosterone levels in male mice. (**B**) Serum corticosterone levels in female mice. Filled circles represent individual mice. Data are the mean ± SEM for *n* = 6–8 mice/group depending on serum volumes. One-way ANOVA with Dunnett’s post hoc test, *p* > 0.05, not significant.

Corticosterone levels were measured in serum of mice after 28 days of PET-MP exposure using an ELISA. No significant changes in corticosterone levels were detected in male or female mice (Figure 8).

### 2.6. Histopathology

**Figure 9:**
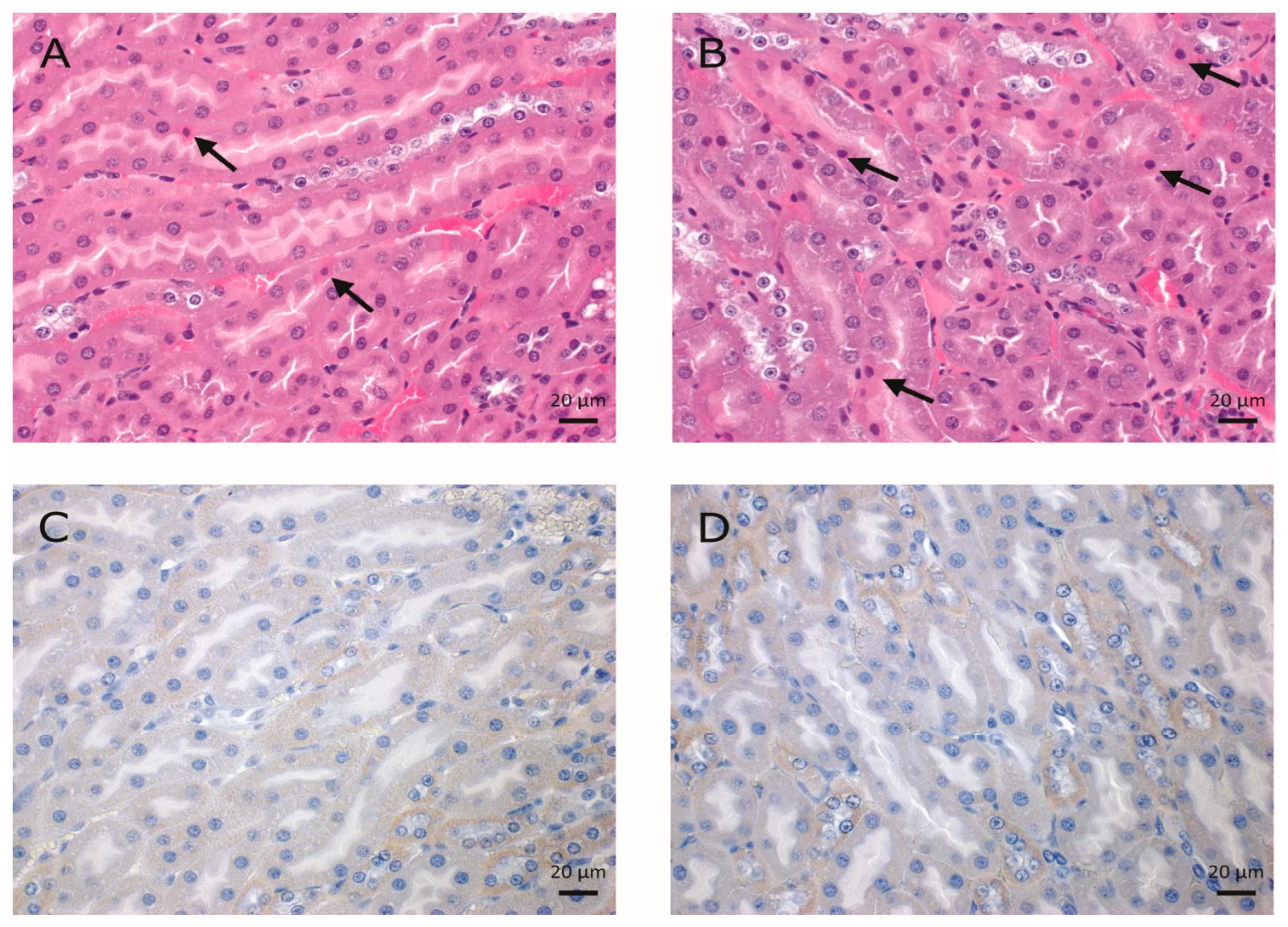
Apoptosis of tubular epithelial cells in the kidneys of male mice. Representative H&E staining of the kidney in (**A**) control mice and (**B**) 7.5 mg/kg PET-MP-exposed mice with apoptotic cells (black arrows). Representative TUNEL staining of the apoptotic region in (**C**) control mice and (**D**) 7.5 mg/kg PET-MP-exposed mice.

Histopathological evaluation on the tissues revealed no changes in heart, lungs, stomach, liver, small intestine, colon, and reproductive organs after PET exposure. However, male mice had pathological changes in the kidneys not seen in the female mice. A mild degree of tubular vacuolation was found in both control and PET-MP-exposed male mice across all dose groups. Additionally, apoptosis of tubular epithelial cells, a background lesion in kidneys, was found more frequently in the males than the females on the outer strip of the outer medulla. One control and three of the four high-dose (37.5 mg/kg) male mice examined showed a minimal degree of apoptosis of the tubular epithelial cells, while the next highest dose (7.5 mg/kg) showed a mild degree of apoptosis in three of the four mice exposed to PET-MPs (Figure 9A,B). However, no dose-related effect was noted. TUNEL staining was done to visualize apoptotic cells; however, the epithelial cells appeared to be in the early stages of apoptosis, as no DNA fragmentation was detected (Figure 9C,D). Females had a minimum degree of apoptosis in only 2 out of the 20 mice examined across the treatment groups, 1 in each of 0.3 and 7.5 mg/kg. Representative images for both sexes at every dose can be found in Appendix Figure A2.

**Figure 10:**
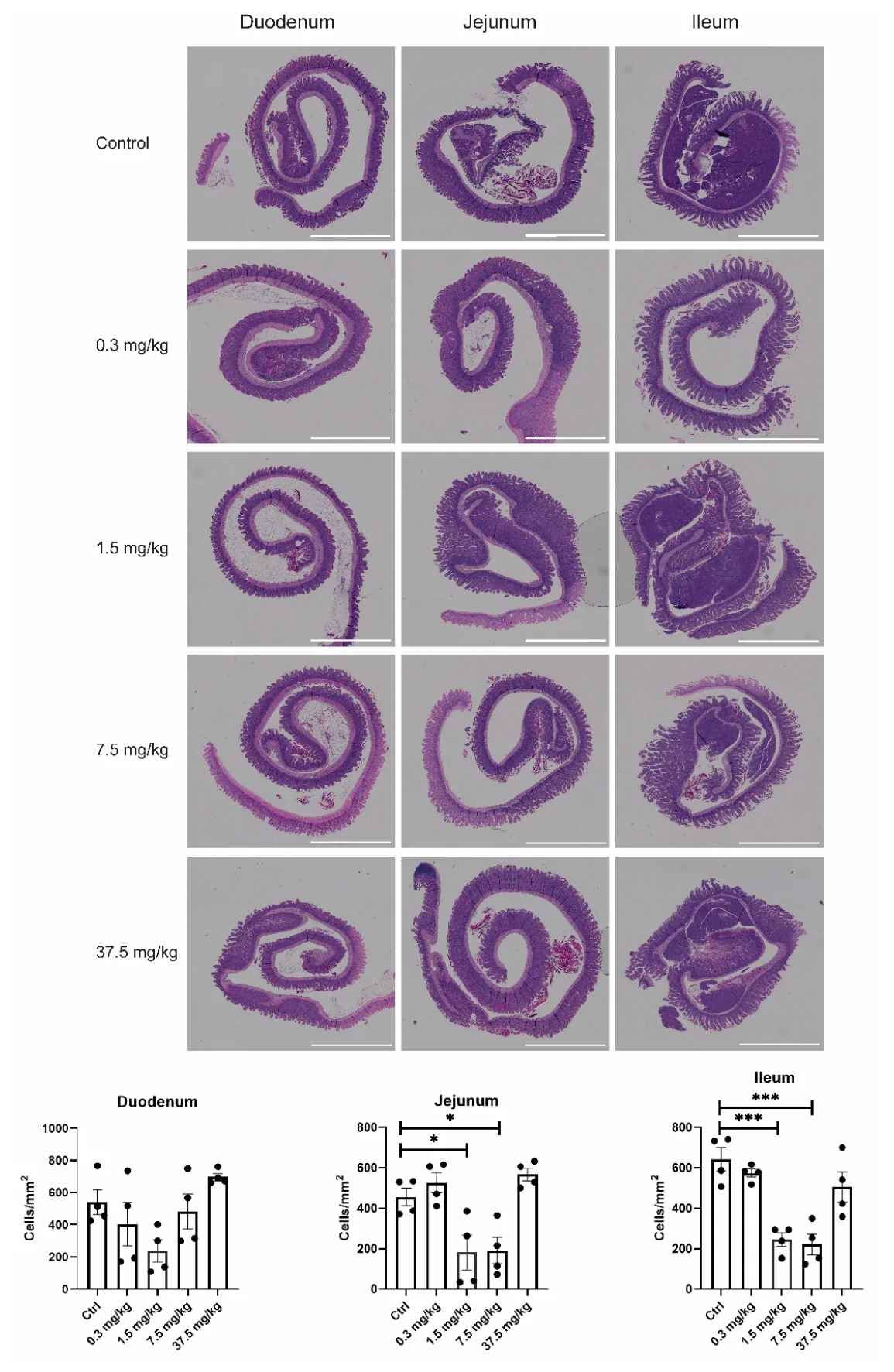
Average goblet cells/area of the three sections of the small intestine of male mice exposed to PET. Goblet cells were PAS stained and enumerated in the duodenum, ileum, and jejunum. Representative images of each section (duodenum, jejunum, and ileum) for every dose are shown. Cell counts were normalized to tissue surface area (mm^2^). Data are the mean ± SEM for *n* = 4 mice/group. Scale bar = 2000 μm. One-way ANOVA with Dunnett’s post hoc test, * *p* ≤ 0.05 and *** *p* ≤ 0.001.

**Figure 11:**
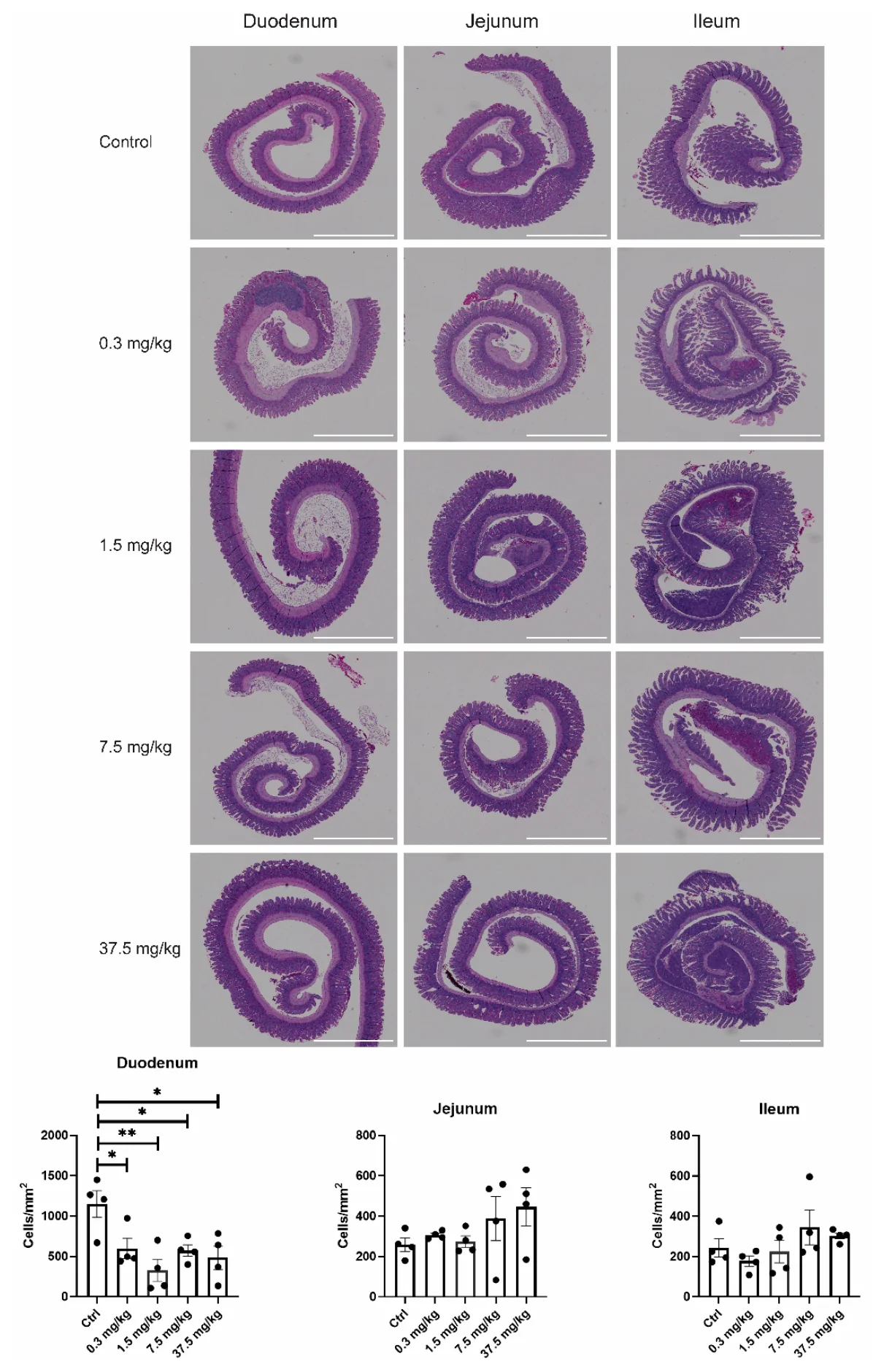
Average goblet cells/area of the three sections of the small intestine of female mice exposed to PET. Goblet cells were PAS stained and enumerated in the duodenum, ileum, and jejunum. Representative images of each section (duodenum, jejunum, and ileum) for every dose are shown. Cell counts were normalized to tissue surface area (mm^2^). Filled circles represent individual mice.Data are the mean ± SEM for *n* = 4 mice/group, except for female 0.3 mg/kg bw/d, *n* = 3. Scale bar = 2000 μm. One-way ANOVA with Dunnett’s post hoc test, * *p* ≤ 0.05 and ** *p* ≤ 0.01.

Although no histopathological changes were seen in the small intestine with H&E, goblet cell populations were examined. In male mice (Figure 10), the two middle doses (1.5 and 7.5 mg/kg bw/d) showed a 3-fold decrease in goblet cells compared to controls in the jejunum and ileum, whereas the goblet cell population in the duodenum of female mice showed a 2-fold decrease at all doses compared to the control (Figure 11).

### 2.7. Small Intestine Permeability

**Figure 12:**
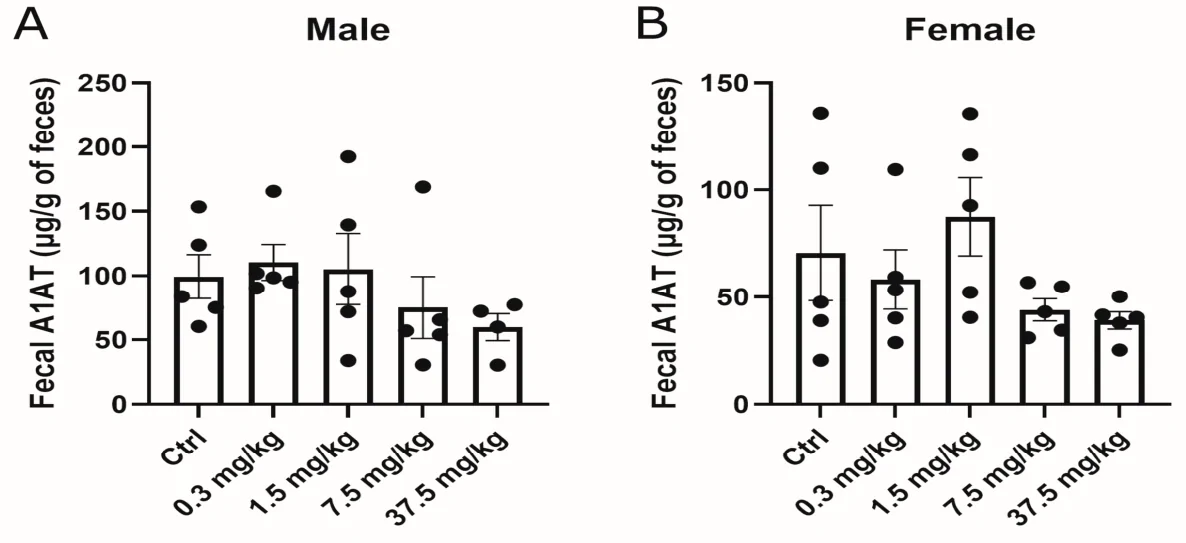
Intestinal permeability not impacted by PET exposure. Alpha-1 antitrypsin (A1AT) was measured in fecal matter collect after 28-day exposure to PET in both (**A**) male and (**B**) female mice. Filled circles represent individual mice. Data are the mean ± SEM for *n* = 5/group, except 37.5 mg/kg bw/d males, as one was below detection limits, *n* = 4. Levels were normalized to total weight of fecal pellet homogenized. Kruskal–Wallis test with Dunn’s post hoc test for males and Welch’s one way ANOVA with Dunnett’s post hoc test for females, *p* > 0.05, not significant.

Intestinal barrier function was indirectly estimated by measuring the levels of alpha 1 antitrypsin (A1AT) in the feces of mice exposed to PET-MPs. A1AT is a protein produced by the liver that circulates in the plasma but leaks across the epithelium during instances of enteric protein loss, which may be associated with epithelial damage. A1AT levels were measured in the feces of mice after 28 days of PET-MP exposure via the ELISA. No changes in AIAT levels were observed at any of the doses in either sex (Figure 12), indicating that PET-MP likely does not induce overt intestinal damage.

## 3. Discussion

Increasing nano- and micro-plastics pollution is of concern due to the potential for human exposure. Humans may be exposed to MNPLs through multiple routes, and the presence of MNPLs in human tissues has been reported [14,15,16,17,18,19,20,21,22,23,24,25,26,27,28,29,30,31,32]. In vivo studies have noted both the accumulation of MNPLs in various organs and the biological effects of MNPL exposure, including oxidative stress, inflammation, and metabolic disorders [4,9,11,47,51]. However, the potential impact of MNPLs on human health remains unknown.

In the environment, MNPLs are of irregular shape, size, and polymer composition [51,53]. PET is one of the most predominant MNPLs in the environment, as it is widely used in consumer goods and packaging, predominantly in food and beverage containers [9,53,54,55,58,59,60]. Studies on the toxicology of environmentally relevant PET-MPs remain limited, and though some studies have investigated the impact of PET-MPs on biological systems, very few have used relevant exposure doses to humans. Furthermore, not only does the size and shape of particles influence MNPL toxicity, but exposure concentration does as well. Many studies use exposure concentrations well above what would be expected in environmental matrices, and more studies examining low-dose PET-MP exposure are warranted to better understand real-world exposure levels and their potential impact on human health.

In this study, we investigated the systemic toxicological effects of PET-MP exposure. Mice were exposed to PET-MPs in a 28-day repeat-dose oral toxicity study, at doses of 0.3, 1.5, 7.5, and 37.5 mg/kg bw/d, which were based on estimated human exposure levels (1.17–22.4 mg/kg bw/d) [51,54,57]. At all four concentrations tested, we observed no changes in bodyweight or food consumption in any of the mice. Similarly, other studies have noted no change in bodyweight or food consumption in mice exposed to PET-MPs [9,51,52,55,61,62,63]. However, other studies did observe changes in the bodyweight of mice after exposure to PET [52,53,54,58,59,64]. Our results also showed no changes in any organ coefficients. However, some studies have noted changes in heart, spleen, and testis weight [51,54,58,61], while others have noted no change in testis or liver weights [52,53,55,63].

We examined clinical biochemistry and hematology of both the male and female mice exposed to PET. A few minor changes were noted in immunocytes of both male and female mice, but only at a singular dose. Changes in immunocyte populations have been noted after exposure to polystyrene MPs [65], but the biological relevance of our hematological data is inconclusive and unlikely to indicate an immune response. No significant differences were noted in the clinical biochemistry tests of either sex. Specifically, the markers we looked at, cholesterol (TC), triglycerides (TG), high-density lipoprotein cholesterol (HDL), low-density lipoprotein cholesterol (LDL), lipase, total bilirubin, blood urea nitrogen, alkaline phosphatase (ALP), and albumin, were used to assess liver and kidney function, as well as metabolic function. Although we detected no changes, other studies have observed changes in biochemical tests in mice exposed to PET. Other studies have noted changes in blood nitrogen urea, glucose, ALT, TG, AST, cholinesterase, and TC levels after PET exposure [51,54,58,66,67,68]. Despite the fact that we did not see any significant changes in the biochemical analysis, the other studies show that PET-MPs may impact liver function. As the primary detoxification organ, the liver is also a primary target for MP toxicity [69,70]. PET-MPs could potentially disrupt energy homeostasis and lipid metabolism, resulting in hepatotoxicity. The discrepancy in our results compared to those in the literature could be due to several factors, including the duration of MP exposure, the doses used, and MP particle size.

MPs have been shown to elicit the production of cytokines, modulate the immune response, and cause inflammation [4,71]. Dysregulation of the immune response can result in immunosuppression or chronic inflammation [4]. In our study, after 28-day exposure to PET-MPs we saw no changes in splenic and thymic immune cell populations in either male or female mice. A few studies have found decreased CD8^+^ T-cell and CD4^+^/CD8^+^ T-cell ratio due to a drop in CD4^+^ T-cell numbers and changes in immune cell populations of mesenteric lymph nodes [69,72,73]. However, there is a distinct lack of in vivo studies that assess the impact of MNPLs on immune cell populations and more research should be carried out to determine how PET exposure may modulate the immune response and impact immune cell populations.

In addition to changes in immune cell populations, an immune response results in the production of cytokines and chemokines. In order to measure systemic inflammation, serum cytokine/chemokine expression was analyzed. Female mice displayed a statistically significant increase in LIX (CXCL5) and IL-17F at the highest dose. These are both pro-inflammatory cytokines involved in neutrophil recruitment [74,75]. Conversely, males displayed a statistically significant decrease in MIP-2 and TNF-α at the highest dose, both pro-inflammatory cytokines involved in neutrophil recruitment and cell activation, respectively [76]. While these results should be interpreted with caution, as a false discovery rate correction indicates that they are likely cases of type I error that arose due to the volume of analytes being compared in our multiplex Luminex assay, it is interesting to note that regression analysis indicated that males showed a decreasing trend in several pro-inflammatory cytokines, while females showed an increasing trend in a non-overlapping subset of pro-inflammatory cytokines. While the magnitude of change in cytokine expression levels after 28-day PET-MP exposure in either males or females would not be enough to drive an inflammatory response in isolation, it is possible that these subtle differences in baseline conditions would influence the course of active immunity, and it would be interesting to determine whether male and female mice exposed to PET diverge in their overall response to experimental models of inflammation or infection. A study by Sun et al. showed similar effects, with modest PET-induced increases in pro-inflammatory IL-5, IL-2, and TNF-α, particularly at higher doses (50 and 250 mg/kg bw/d), and a decrease in anti-inflammatory IL-10 [54]. These changes were observed both systemically and locally. Inflammation may still change locally, but systemically no excessive changes were noted in cytokine levels. Also, no changes in serum corticosterone levels were observed in either male or female mice after exposure to PET-MPs, a glucocorticoid involved in the stress response, which can modulate cytokine expression [77]. Overall, our results did not indicate an inflammatory response or suppression of an immune response after PET-MP exposure for 28 days. However, longer exposure durations should be explored in future research to investigate whether changes in systemic inflammation may be present.

The gastrointestinal tract is the first organ exposed to MNPLs upon ingestion. PET-MPs could act as foreign objects causing decreased mucin secretion and impairment of the intestinal barrier function [54,78]. Goblet cells are the main producer of mucus in the intestine, which acts as a protective layer lining the intestinal wall and contributes to maintaining intestinal barrier function [79]. Histological analysis of the PET-exposed mice showed no pathological changes nor inflammatory cell infiltration in the small intestine or colon. However, we did observe a change in PAS-positive goblet cells in the jejunum and ileum of male mice at the two middle doses, but not in a dose-dependent manner. We also saw decreased PAS-positive goblet cells in the duodenum of female mice at all doses, with the magnitude of decrease similar across the dose range. A study by Harusato et al. noted no changes in histology or mucus thickness in the colon of mice exposed to PET-MPs for eight weeks, and a corresponding lack of effect on goblet cell populations [79]. However, at very high-dose PET-NP exposure, cysts and inflammatory lesions developed in the intestine of mice [59]. The decrease in goblet cell populations can be an early sign of an inflammatory process, wherein mucosal changes happen before inflammation [80]. Perhaps longer exposure to PET-MPs would result in decreased mucus in the intestine, immune cell infiltration, and increased intestinal permeability. We investigated enteric protein loss using A1AT presence in feces as a surrogate marker of intestinal damage. A1AT is a glycoprotein primarily produced in the liver and circulates in the bloodstream, but can pass from the bloodstream to the intestine when the intestinal barrier is weakened or damaged [81,82]. After 28 days of PET exposure, no change in enteric protein loss was noted at any dose for either sex, indicating that PET exposure does not induce overt epithelial damage. It should be noted, however, that a lack of PET-induced changes for this marker does not rule out subclinical changes in intestinal permeability, and future studies should include a sensitive tracer method for gut permeability, such as Cr51-EDTA or FITC-dextran, to assess this with greater confidence.

The liver and kidneys play a large role in the detoxification of xenobiotics [83]. A small proportion of consumed MPs may be able to translocate through the gut and enter systemic circulation, dispersing to other organs [71,83], and MNPLs smaller than 10 µm may be able to translocate into the cell [9,53]. MPs have been detected in the liver and kidneys of mice after oral consumption [3,36,83]. In the primary detoxification organs, including the liver and kidney, histological analysis did not reveal any changes in the liver, but mild apoptosis was noted in the kidney of male mice with low severity and low incidence. Furthermore, there were no histological changes in the lungs, stomach, heart, or reproductive organs. Several other studies on PET-MP exposure have noted histological changes in the heart, liver, kidney, and lungs [9,51,52,58,59,61,66,67,68], with the predominant change being inflammatory cell infiltration. MPs can result in changes in cell morphology, apoptosis, and autophagy of the epithelial cells of kidney tubules [84]. Despite the presence of mild apoptosis in tubular epithelial cells of the outer strip of the outer medulla, no inflammatory cell infiltration was noted in the kidneys. Combined with the lack of changes in urea nitrogen and bilirubin, our results indicate that there is no acute nephrotoxicity from MP exposure, due to the low severity and lack of dose response across PET exposure groups. Future research should explore whether any renal effects are observed following longer exposure durations with PET microplastics.

MP particles in the environment are exposed to natural elements, which degrade the particles, resulting in fragmentation and particles that are of irregular size and shape [51,85]. Yet, many toxicological studies have been performed using particles of uniform size and regular shape [36,39,40,42,44,86,87]. In this study, we used PET-MPs of irregular shape in various sizes to better represent potential real-world exposure. It is becoming more evident that the physiochemical properties of MP particles alter particle uptake and their toxicity [9,60,88,89,90]. Not only do size, shape, and polymer composition impact toxicity, exposure concentration and duration may also play a role [51,60]. The use of PET-MPs with different shapes and size, as well as exposure concentrations and lengths, throughout the literature contribute to the variability seen in the results.

The findings of this study demonstrate that low-dose, short-term exposure to PET-MPs does not promote systemic toxicity or an inflammatory response. A few limitations to our study include the size range of particles, lack of bioaccumulation data, and short exposure time. Bioaccumulation data would be helpful in determining if the PET-MPs used in this study are able to translocate through the intestinal epithelium and amass in other organ tissues. Without these data it is difficult to determine whether the absence of adverse outcomes observed in the mice used in this study is due to a lack of toxicity or a lack of genuine exposure to the body proper. The observation of bioaccumulation over the course of a longer OECD guideline-compliant study, such as the 90-day TG408, would be beneficial in determining potential PET toxicity and better represent chronic exposure that can be extrapolated to human toxicity. Additionally, smaller particles < 1.5 µm and into the nanoplastic range are thought to be able to penetrate organs, and studies using these sized particles may inform potential PET-MP toxicity. With this in mind, it should be noted the particles used in this study were distributed in size, ranging from less than 1.5 µm to greater than 11 µm. While we took care to ensure that each dosing solution was prepared identically, variations in this distribution between dosing preparations could conceivably result in altered responses between mice and interfere with the reproducibility of toxicological parameters. Therefore, more research on PET NPs and MPs is necessary and critical for regulatory risk assessment.

## 4. Materials and Methods

### 4.1. Particle Characterization

PET powder was purchased from Nanochemazone (NCZ-PA-138/24; Leduc, AB, Canada). The size and morphology of the particles were confirmed by scanning electron microscope (TESCAN, Brno, Czech Republic). The zeta-potential was measured using a dynamic light scattering (DLS) instrument (Zetasizer PN3702, Malvern, UK). PET particles were tested for endotoxin contamination using a E-TOXATE kit (MilliporeSigma, Milwaukee, WI, USA).

### 4.2. Animal Care and Treatment

One hundred 9-week-old C57BL/6 mice (fifty male and fifty female) were purchased from Charles River (Saint-Constant, QC, Canada). Prior to dosing, mice were acclimatized to the animal housing facility for at least a week. Mice were randomized into 5 treatment groups of 10/sex, marked with a tail tattoo, and housed individually (males) or pair-housed (females). Animal handling and treatment were conducted according to the Canadian Council of Animal Care and were approved by the Health Canada Animal Care Committee (ACC: 2025-007, Date: 4 December 2025, Ottawa, ON, Canada). Mice were on a 12 h light/dark cycle and food (Teklad 2014 pelleted diet; Inotiv, Madison, WI, USA) and water were provided ad libitum. Mice received daily gavage with either vehicle (water with 0.01% Tween-20 (MilliporeSigma)) or PET particles at doses of 0.3, 1.5, 7.5, or 37.5 mg/kg per bodyweight/day over a 28-day period, using the OECD 407 repeated-dose 28-day oral toxicity study protocol. Prior to gavage, animals were weighed daily. Food consumption by singly caged males or co-housed female pairs was measured on a weekly basis, and the total consumption was converted to a daily mean over the course of the study per individual mouse. Daily monitoring of general health was conducted. At study termination, a full necropsy was done on each mouse. Total cumulative dose of plastic was calculated for each animal and averaged per dose group (Appendix Table A1).

### 4.3. Dosing Solution Preparation

The solutions were prepared by mixing powdered PET particles (Nanochemazone) in 0.01% Tween-20 water. The solution was then sonicated in a water bath (Branson CPX2800; Emerson, St. Louis, MO, USA) for 2 rounds of 30 min at 40 kHz. Subsequent dosing solutions were made by diluting the sonicated solution to the corresponding doses in Tween-20 water. All dosing solutions were sonicated for a final time at 40 kHz for 30 min.

### 4.4. Tissue Collection

A complete gross examination was performed on all mice after animals were euthanized by exsanguination via abdominal aortic puncture under isoflurane anesthesia (Baxter, Mississauga, ON, Canada). Heart, liver, lungs, spleen, thymus, thyroid, kidneys, adrenals, stomach, small intestine, colon, brain, and both male and female reproductive organs were collected, weighed, and either fixed for histopathological analysis (*n* = 4/group/sex) or frozen for further analysis (*n* = 6/group/sex). For histopathology, tissues were fixed in 10% neutral buffered formalin (FisherScientific, Edmonton, AB, Canada), except testes, which were immersion-fixed in Bouin’s solution (VWR, Secaucus, NJ, USA). Spleen and thymus were collected for immunophenotyping (*n* = 6/group/sex), and blood was collected for clinical biochemistry in SST microtainer tubes (Becton Dickinson (BD), Franklin Lakes, NJ, USA) and hematology in K_2_EDTA microtainer tubes (BD) (*n* = 10/group/sex).

### 4.5. Histology

After fixation, paraffin-embedded tissues were cut in 4 µm sections and slides were stained with SelecTech hematoxylin and eosin (H&E; Leica Biosystems, Concord, ON, Canada).

For the small intestine, 2 cm-long segments of the duodenum, jejunum, and ileum were Swiss-rolled prior to fixation and subsequently cut at 3 µm, deparaffinized, and stained with periodic acid-Schiff–hematoxylin (PAS; MilliporeSigma, Saint Louis, MO, USA), Surgipath SelecTech Hematoxylin 560 MX, Define MX-aq, and Blue Buffer 8 (Leica Biosystems) to identify goblet cells. Goblet cells were enumerated by counting the PAS-positive cells in one section per intestinal segment (duodenum, ileum, and jejunum) and then transforming the count value over sectional surface area (mm^2^) in ImageJ (1.52p; NIH, Bethesda, MD, USA).

TUNEL staining was performed according to the manufacturer’s instructions using the TUNEL Assay kit (AB206386 Abcam, Waltham, MA, USA) on 4 µm kidney sections. In brief, permeabilization using Proteinase K (1:100 in distilled water) for 20 min was done after deparaffinization in toluene and rehydration in graded alcohols. Inactivation of endogenous peroxidases was done for 5 min using 3% H_2_O_2_ in methanol. TdT labeling was done following incubation with equilibration buffer, by incubating TdT enzyme in TdT labeling reaction mix. Reaction was terminated with stop buffer. Blocking buffer was used to minimize non-specific binding. Signal detection and development were done using a 1:25 dilution of conjugate followed by a 1:30 working solution of DAB solution 1 and 2. Counterstaining was performed by dipping 2× in Leica SelecTech Hematoxylin 560 MX and then rinsing in distilled water, followed by cover-slipping in aqueous mounting media. Then, 20 mM Tris-buffered saline was used as rinse buffer for all steps.

### 4.6. Hematology, Serum Biochemical Analysis, Multiplexing, and Corticosterone ELISA

Red blood cell (RBC) count, hematocrit (HCT), hemoglobin (HGB), mean corpuscular volume (MCV), mean corpuscular hemoglobin (MCH), mean corpuscular hemoglobin concentration (MCHC), platelet counts (PTL), mean platelet volume (MPV), white blood cell (WBC) count, neutrophils, monocytes, basophils, and eosinophils were counted in whole blood using a XT2000i Automated Hematology Analyzer (Sysmex, Lincolnshire, IL, USA).

Serum was separated by centrifugation at 1000× *g* for 10 min at room temperature. The following parameters were analyzed using a Dimension EXL 200 Integrated Chemistry System and reagents (Siemens Health Care Limited, ON, Canada): cholesterol (mmol/L), triglycerides (mmol/L), HDL cholesterol (mmol/L), LDL cholesterol (mmol/L), lipase (U/L), total bilirubin (µmol/L), urea (mmol/L), alkaline phosphatase (U/L), and albumin (g/L). Serum cytokines and chemokines were analyzed using a 41-plex multiplex kit (MilliporeSigma) on a Luminex 200 (Diasorin, Saluggia, Italy). Detection limits were set within a standard curve generated according to the kits’ protocol.

Serum corticosterone was measured using the DetectX^®^ Corticosterone ELISA kit (Arbor Assays, Ann Arbor, MI, USA) according to the manufacturer’s instructions. In short, serum samples were pre-treated with a dissociation reagent to release protein-bound corticosterone, allowing measurement of the total corticosterone from the samples.

### 4.7. Immunophenotyping

**Figure A1:**
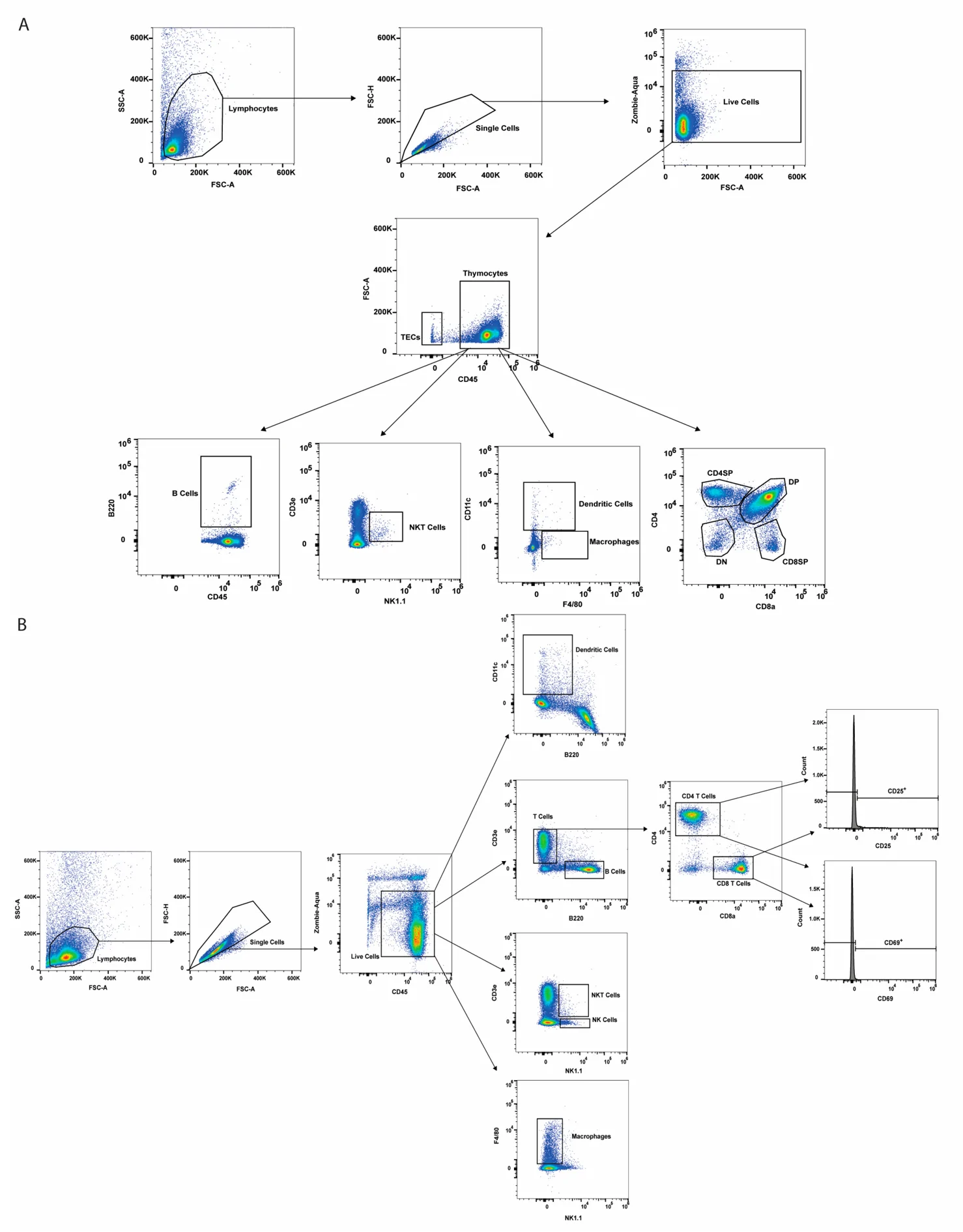
Flow cytometry gating strategy for immunophenotyping. (**A**) Thymus and (**B**) spleen.

**Figure A2:**
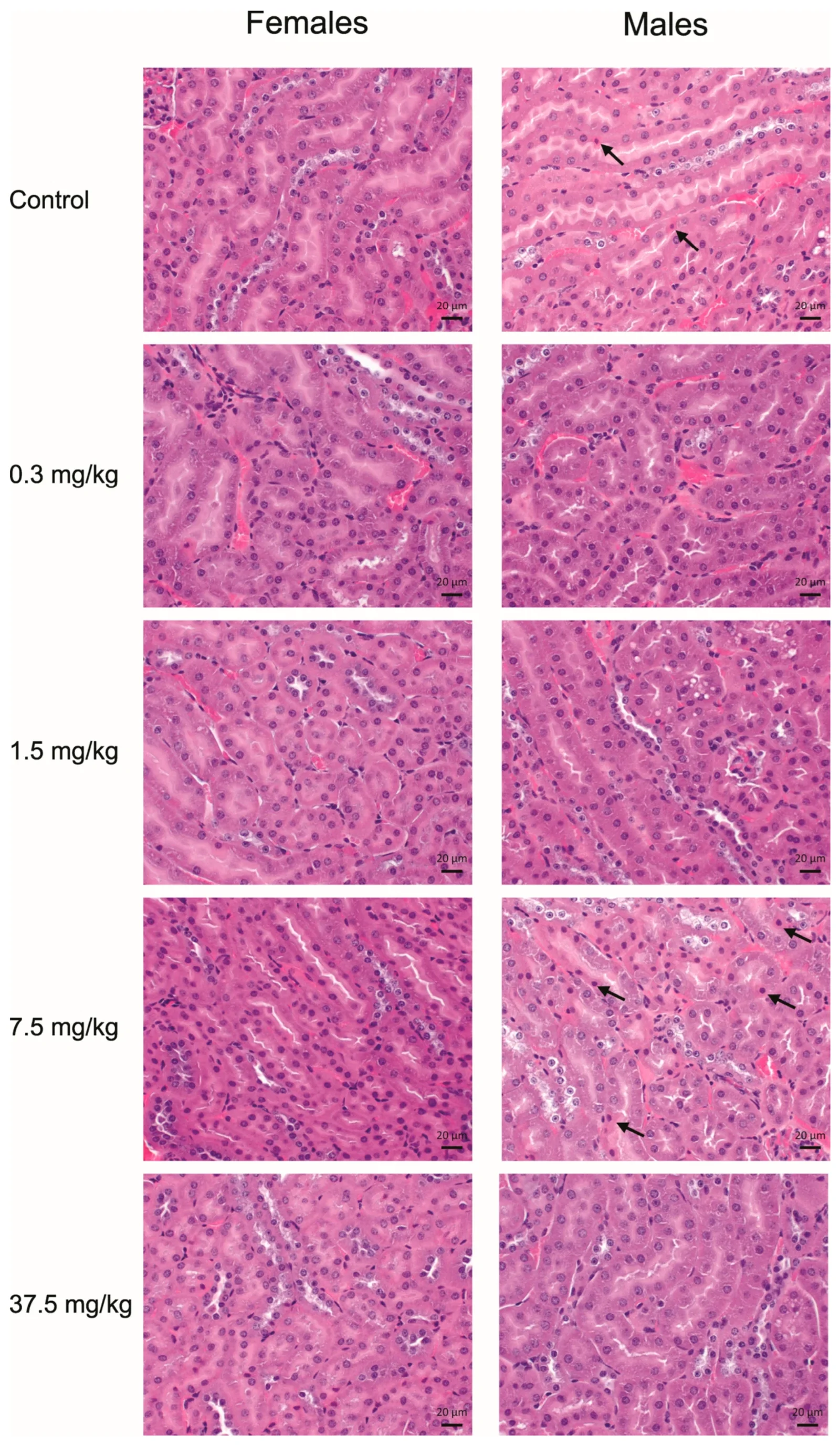
Representative H&E staining of the kidney in male and female PET-MP-exposed mice. Arrows denote apoptotic bodies.

Isolation of thymocytes and splenocytes was conducted by gently crushing tissue in a 70 µm nylon cell strainer (Fisher Scientific, Whitby, ON, Canada) and rinsing into a 50 mL conical tube with DMEM media (Wisent, St-Bruno, QC, Canada) supplemented with 10% FBS (Wisent). Erythrocytes were removed from splenocyte suspensions using RBC lysis buffer (ThermoFisher, Burlington, ON, Canada). Subsequently, cells were counted, and Fc block (BD) was added and surface stained using the following antibodies: B220-BV786, CD11c-BV421, CD45-RB705, CD3ε-FITC, CD25-PeCy7, F4/80-RV610, CD69-PE, CD8α-R718, NK1.1-BUV737, CD4-BUV395 (BD), and Zombie Aqua fixable viability dye (Biolegend, San Diego, CA, USA). Analysis of cell populations was done using a spectral flow cytometer, Sony ID7000E (Sony Biotechnology, San Jose, CA, USA), and FlowJo analysis software (v10.9, BD). Appendix Figure A1 shows a representative gating strategy. Doublets, debris, and dead cells were gated out. The remaining cells were sorted by CD45. Immune cell populations were divided into B-cells and T-cells and then further subdivided into CD4^+^ or CD8^+^ T-cells. Activation markers CD69 and CD25 were used to analyze general immune cell and T-cell-specific expression. Natural killer (NK) cells were identified as NK1.1^+^ cells, and NKT cells were identified as CD3^+^ and NK1.1^+^ double-positive cells. Macrophages and dendritic cells were identified via F4/80 and CD11c staining, respectively.

### 4.8. Alpha-1 Antitrypsin ELISA

Fecal pellets were collected on the day of necropsy and snap frozen. Fecal pellets were weighed and homogenized for 20 s in a 0.1% Tween-20 solution using a bead-mill homogenizing device (Omni International, Kennesaw, GA, USA). Homogenates were then pelleted by centrifugation for 10 min at 12,000× *g* and supernatant was assessed for alpha-1 antitrypsin (A1AT) by ELISA (Abcam). A1AT concentrations were normalized to the mass of the fecal pellet.

### 4.9. Statistical Analysis

Statistical comparisons were performed using GraphPad (v10.8; Dotmatics, Boston, MA, USA). Data were analyzed for normality using the Shapiro–Wilk test and equal variance was tested using Bartlett’s test. Multiple group comparisons were conducted on data from each dose group by one-way analysis of variance (ANOVA) for equal variance or Welch’s ANOVA for unequal variance, followed by post hoc pairwise comparison using Dunnett’s test. Multiple group comparisons of non-parametric data were conducted using the Kruskal–Wallis ANOVA followed by Dunn’s post hoc pairwise comparison. Two-way ANOVA was used to assess bodyweight changes over time. Multiplex Luminex cytokine analysis was corrected for false discovery rate (FDR) using the Benjamini–Hochberg procedure. Dose–response trends in cytokine expression were analyzed using the Pearson correlation coefficient for parametric data and Spearman rank correlation coefficient for non-parametric data.

## 5. Conclusions

As an extensively used plastic polymer in consumer goods, PET is one of the most predominant MNPLs in the environment. Several types of microplastics, including PET, have been found in human biological fluids and tissues. Yet the potential impact of PET exposure on human health remains unknown. In this study, mice exposed to PET-MPs for 28 days showed no overt systemic toxicity. However, PET exposure altered histology of the kidney and small intestine in a sex-specific manner, but no nephrotoxicity or changes in gut permeability were observed (Table 9). A longer exposure study may further reveal the full impact of PET-MPs on the potential nephrotoxicity and gastrointestinal alterations. Our study highlights how environmentally relevant, low-dose 28-day repeated exposure of PET-MPs results in minimal systemic toxicity. Nevertheless, significant knowledge gaps remain in MNPL research, which include human exposure levels, the threshold level that may be associated with potential health effects, and possible long-term health implications.

## Figures and Tables

**Table 1:** Organ weights in male mice exposed to PET-MPs.

			PET (mg/kg/d)		
	0	0.3	1.5	7.5	37.5
Final bw	27.12 ± 0.54	27.02 ± 0.66	27.29 ± 0.60	26.58 ± 0.47	26.92 ± 0.79
Liver (/bw)	0.0528 ± 0.0022	0.0521 ± 0.0006	0.0526 ± 0.0008	0.0524 ± 0.0007	0.0514 ± 0.0006
Kidneys (/bw)	0.0122 ± 0.0002	0.0123 ± 0.0003	0.0126 ± 0.0003	0.0122 ± 0.0003	0.0127 ± 0.0002
Spleen (/bw)	0.0024 ± 0.0001	0.0024 ± 0.0000	0.0024 ± 0.0000	0.0025 ± 0.0001	0.0025 ± 0.0001
Heart (/bw)	0.0050 ± 0.0001	0.0052 ± 0.0001	0.0051 ± 0.0001	0.0052 ± 0.0001	0.0051 ± 0.0001
Lungs (/bw) ^a^	0.0083 ± 0.0004	0.0080 ± 0.0004	0.0085 ± 0.0004	0.0081 ± 0.0004	0.0082 ± 0.0002
Thymus (g)	0.048 ± 0.002	0.050 + 0.002	0.052 ± 0.003	0.052 ± 0.002	0.054 ± 0.003
Epidydimides (g)	0.110 ± 0.005	0.111 ± 0.009	0.186 ± 0.072	0.096 ± 0.005	0.108 ± 0.09
Testes (g)	0.203 ± 0.005	0.199 ± 0.007	0.193 ± 0.015	0.178 ± 0.010	0.194 ± 0.007
Seminal vesicles, coagulating glands, and prostate (/bw)	0.0154 ± 0.0004	0.0155 ± 0.0003	0.0162 ± 0.0004	0.0161 ± 0.0004	0.0163 ± 0.0003
Stomach (/bw) ^a^	0.0078 ± 0.0005	0.0078 ± 0.0002	0.0082 ± 0.0004	0.0078 ± 0.0004	0.0076 ± 0.0005
Small intestine (/bw) ^a^	0.0431 ± 0.0013	0.0415 ± 0.0009	0.0434 ± 0.0018	0.0401 ± 0.0012	0.0398 ± 0.0008
Colon (/bw) ^a^	0.0082 ± 0.0004	0.0079 ± 0.0005 ^b^	0.0082 ± 0.0004	0.0074 ± 0.0003 ^b^	0.0079 ± 0.0004
Brain (/bw) ^a^	0.0161 ± 0.0004	0.0161 ± 0.0003	0.0163 ± 0.0005	0.0156 ± 0.0006	0.0163 ± 0.0003

Data are expressed as mean ± standard error of the mean for 10 mice/group with the following exceptions: lungs, stomach, small intestine, colon, and brain, *n* = 6 (^a^), except (^b^) *n* = 4, as 2 colons were not weighed.

**Table 2:** Organ weights in female mice exposed to PET-MPs.

			PET (mg/kg/d)		
	0	0.3	1.5	7.5	37.5
Final bw	21.90 ± 0.53	22.58 ± 0.48	21.90 ± 0.37	21.94 ± 0.51	22.36 ± 0.54
Liver (/bw)	0.0491 ± 0.0010	0.0481 ± 0.0007	0.0466 ± 0.0013	0.0472 ± 0.0009	0.0492 ± 0.0006
Kidneys (/bw)	0.0110 ± 0.0002	0.0106 ± 0.0002	0.0113 ± 0.0005	0.0105 ± 0.0002	0.0109 ± 0.0002
Spleen (/bw)	0.0037 ± 0.0002	0.0034 ± 0.0001	0.0038 ± 0.0002	0.0036 ± 0.0001	0.0036 ± 0.0002
Heart (/bw)	0.0053 ± 0.0001	0.0054 ± 0.0001	0.0054 ± 0.0001	0.0053 ± 0.0001	0.0053 ± 0.0001
Lungs (/bw) ^a^	0.0093 ± 0.0005	0.0092 ± 0.0005 ^b^	0.0092 ± 0.0004	0.0093 ± 0.0004	0.0085 ± 0.0004
Thymus (g)	0.0658 ± 0.0041	0.0715 + 0.0051	0.0708 ± 0.0015	0.0732 ± 0.0035	0.0695 ± 0.0034
Ovaries (g)	0.0231 ± 0.0014	0.0260 ± 0.0042	0.0230 ± 0.0008	0.0307 ± 0.0091	0.024 ± 0.0006
Uterus (/bw)	0.0052 ± 0.005	0.0056 ± 0.0003	0.0050 ± 0.0014	0.0054 ± 0.0006	0.0055 ± 0.0004
Stomach (/bw) ^a^	0.0091 ± 0.0004	0.0090 ± 0.0002	0.0102 ± 0.0003	0.0101 ± 0.0003	0.0096 ± 0.0005
Small intestine (/bw) ^a^	0.0499 ± 0.0008	0.0492 ± 0.0006	0.0505 ± 0.0014	0.0495 ± 0.0013	0.0492 ± 0.0006
Colon (/bw) ^a^	0.0108 ± 0.0008	0.0102 ± 0.0006	0.0105 ± 0.0007	0.0101 ± 0.0007	0.0106 ± 0.0006
Brain (/bw) ^a^	0.0207 ± 0.0003	0.0204 ± 0.0003	0.0203 ± 0.0003	0.0203 ± 0.0006	0.0206 ± 0.0002

Data are expressed as mean ± standard error of the mean for 10 mice/group with the following exceptions: lungs, stomach, small intestine, colon, and brain *n* = 6 (^a^), except (^b^) *n* = 5.

**Table 3:** Biochemical parameters in male mice exposed to PET-MPs.

			PET (mg/kg/d)		
	0	0.3	1.5	7.5	37.5
Cholesterol (mmol/L)	3.13 ± 0.06	2.97 ± 0.08	3.07 ± 0.12	2.96 ± 0.11	3.16 ± 0.11
Triglycerides (mmol/L)	1.10 ± 0.06	1.38 ± 0.06	1.00 ± 0.04	1.11 ± 0.06	1.03 ± 0.07
HDL (mmol/L)	2.63 ± 0.05	2.48 ± 0.06	2.62 ± 0.08	2.51 ± 0.08	2.67 ± 0.08
LDL (mmol/L)	0.15 ± 0.01	0.13 ± 0.0	0.14 ± 0.01	0.14 ± 0.01	0.15 ± 0.01
Lipase (U/L)	35.70 ± 0.75	43.10 ± 5.04	38.30 ± 1.58	34.50 ± 1.34	36.00 ± 2.37
Total Bilirubin (µmol/L)	6.33 ± 0.38	6.88 ± 0.54	7.08 ± 0.47	7.10 ± 0.46	5.98 ± 0.55
Urea Nitrogen (mmol/L)	7.64 ± 0.33	8.13 ± 0.39	7.48 ± 0.25	7.28 ± 0.22	6.86 ± 0.27
Alkaline Phosphatase (U/L)	78.50 ± 2.41	76.60 ± 2.03	86.10 ± 6.86	75.30 ± 1.73	74.70 ± 1.85
Albumin (g/L)	13.17 ± 0.23	13.15 ± 0.13	13.31 ± 0.24	12.99 ± 0.19	13.37 ± 0.18

Data are expressed as mean ± standard error for *n* = 10 mice/group.

**Table 4:** Biochemical parameters in female mice exposed to PET-MPs.

			PET (mg/kg/d)		
	0	0.3	1.5	7.5	37.5
Cholesterol (mmol/L)	2.27 ± 0.05	2.39 ± 0.06	2.36 ± 0.09	2.39 ± 0.12	2.32 ± 0.07
Triglycerides (mmol/L)	0.58 ± 0.04	0.63 ± 0.04	0.57 ± 0.05	0.61 ± 0.04	0.62 ± 0.04
HDL (mmol/L)	1.88 ± 0.03	1.97 ± 0.05	1.93 ± 0.09	2.01 ± 0.09	1.94 ± 0.06
LDL (mmol/L)	0.26 ± 0.02	0.28 ± 0.02	0.30 ± 0.01	0.28 ± 0.02	0.27 ± 0.02
Lipase (U/L)	31.70 ± 1.18	30.60 ± 0.99	29.80 ± 0.61	32.60 ± 0.85	31.00 ± 1.46
Total Bilirubin (µmol/L)	6.58 ± 0.63	5.66 ± 0.58	6.22 ± 0.53	7.54 ± 0.67	6.84 ± 0.60
Urea Nitrogen (mmol/L)	5.58 ± 0.31	5.60 ± 0.29	5.73 ± 0.33	5.57 ± 0.29	5.52 ± 0.24
Alkaline Phosphatase (U/L)	128.20 ± 2.55	131.30 ± 3.05	128.90 ± 2.50	137.40 ± 2.77	133.00 ± 6.05
Albumin (g/L)	14.64 ± 0.23	14.91 ± 0.22	14.78 ± 0.19	15.17 ± 0.19	14.70 ± 0.15

Data are expressed as mean ± standard error for *n* = 10 mice/group.

**Table 5:** Hematology parameters in male mice exposed to PET-MPs.

			PET (mg/kg/d)		
	0	0.3	1.5	7.5	37.5
WBCs (10^9^/L)	2.92 ± 0.38	4.35 ± 066	2.93 ± 0.66	2.72 ± 0.49	2.74 ± 0.27
RBCs (10^12^/L)	9.92 ± 0.11	9.99 ± 0.12	10.12 ± 0.08	10.02 ± 0.09	10.26 ± 0.06
HGB (g/L)	141.00 ± 1.33	142.30 ± 1.69	143.70 ± 0.93	142.50 ± 1.65	145.30 ± 0.88
HCT (L/L)	0.467 ± 0.004	0.469 ± 0.004	0.471 ± 0.003	0.472 ± 0.004	0.474 ± 0.002
MCV (fL)	47.07 ± 0.14	46.95 ± 0.12	46.59 ± 0.20	47.08 ± 0.13	46.26 ± 0.16
MCH (pg)	14.22 ± 0.06	14.24 ± 0.02	14.19 ± 0.04	14.22 ± 0.08	14.17 ± 0.04
MCHC (g/L)	302.20 ± 0.71	303.40 ± 0.95	304.90 ± 0.90	302.10 ± 1.23	306.30 ± 0.79
PTL (10^9^/L)	1133.00 ± 101.87	1202.40 ± 52.93	1239.10 ± 50.13	1176.00 ± 37.95	1079.10 ± 103.32
MPV (fL)	6.48 ± 0.06	6.45 ± 0.06	6.46 ± 0.02	6.50 ± 0.04	6.50 ± 0.05
Neutrophils (10^9^/L)	0.23 ± 0.03	0.40 ± 0.06 *	0.24 ± 0.04	0.26 ± 0.04	0.29 ± 0.02
Lymphocytes (10^9^/L)	2.58 ± 0.34	3.77 ± 0.62	2.57 ± 0.62	2.36 ± 0.45	2.34 ± 0.25
Monocytes (10^9^/L)	0.05 ± 0.02	0.10 ± 0.02	0.07 ± 0.02	0.04 ± 0.01	0.06 ± 0.01
Eosinophils (10^9^/L)	0.05 ± 0.01	0.08 ± 0.02	0.05 ± 0.01	0.05 ± 0.02	0.05 ± 0.01

Data are expressed as mean ± standard error for *n* = 10 mice/group. One-way ANOVA with Dunnett’s post hoc test, * *p* ≤ 0.05.

**Table 6:** Hematology parameters in female mice exposed to PET-MPs.

			PET (mg/kg/d)		
	0	0.3	1.5 ^a^	7.5	37.5
WBCs (10^9^/L)	1.50 ± 0.27	2.22 ± 0.25	2.61 ± 0.53	2.04 ± 0.27	2.92 ± 0.49 *
RBCs (10^12^/L)	9.58 ± 0.10	9.59 ± 0.08	9.55 ± 0.14	9.71 ± 0.07	9.64 ± 0.10
HGB (g/L)	137.70 ± 1.32	138.90 ± 1.07	138.67 ± 2.15	140.00 ± 1.02	140.00 ± 1.52
HCT (L/L)	0.453 ± 0.004	0.453 ± 0.003	0.454 ± 0.005	0.458 ± 0.003	0.458 ± 0.005
MCV (fL)	47.31 ± 0.11	47.28 ± 0.22	47.52 ± 0.16	47.17 ± 0.17	47.49 ± 0.13
MCH (pg)	14.37 ± 0.05	14.49 ± 0.04	14.52 ± 0.06	14.43 ± 0.06	14.51 ± 0.03
MCHC (g/L)	303.80 ± 0.93	306.60 ± 1.32	305.56 ± 1.33	306 ± 0.83	305.80 ± 0.92
PTL (10^9^/L)	964.40 ± 6.68	974.40 ± 22.12	822.67 ± 76.95	854.80 ± 122.84	899.70 ± 93.31
MPV (fL)	6.60 ± 0.06	6.62 ± 0.07	6.70 ± 0.06	6.58 ± 0.07 ^a^	6.66 ± 0.09
Neutrophils (10^9^/L)	0.20 ± 0.03	0.25 ± 0.03	0.28 ± 0.04	0.18 ± 0.02	0.27 ± 0.04
Lymphocytes (10^9^/L)	1.18 ± 0.21	1.86 ± 0.23	2.16 ± 0.46	1.72 ± 0.23	2.45 ± 0.42 *
Monocytes (10^9^/L)	0.06 ± 0.03	0.05 ± 0.01	0.09 ± 0.02	0.08 ± 0.03	0.12 ± 0.03
Eosinophils (10^9^/L)	0.06 ± 0.03	0.05 ± 0.04	0.07 ± 0.02	0.05 ± 0.01	0.08 ± 0.02

Data are expressed as mean ± standard error for *n* = 10 mice/group with the following exception: *n* = 9 (^a^). One-way ANOVA with Dunnett’s post hoc test, * *p* ≤ 0.05.

**Table 7:** Dose-dependent trends in male serum cytokines after PET-MP exposure.

	**Males**	
**Cytokine**	**r**	***p*** ** -Value**
IL-1β	−0.4813	0.0071
IL-6	−0.5581	0.0025
IL-7	−0.4926	0.0057
IL-12p40	−0.4686	0.0158
MIP-1α	−0.4936	0.0056
MIP-2	−0.485	0.0066
TNF-α	−0.6208	0.0003

**Table 9:** Summary of study findings.

Outcome	Effect of PET on Males	Effect of PET on Females
Food Intake	No effect	No effect
Bodyweight	No effect	No effect
Biochemistry	No effect	No effect
Hematology	Neutrophils elevated at 0.3 mg/kg	WBC and lymphocytes elevated at 37.5 mg/kg
Immunocyte Populations	No effect	No effect
Plasma Pro-inflammatory Cytokines	Decreasing trend in IL-1β, IL-6, IL-7, IL-12p40, MIP-1α, MIP-2, and TNF-α	Increasing trend in Eotaxin, IL-1α, IL-17F, and LIX
Corticosterone	No effect	No effect
Histopathology	3-fold decrease in goblet cells at 1.5 and 7.5 mg/kg bw/d compared to controls in the jejunum and ileum	2-fold decrease in goblet cells at all doses compared to controls in the duodenum

## Data Availability

Data will be made available upon request.

## Abbreviations

The following abbreviations are used in this manuscript:

MP	Microplastic
NP	Nanoplastic
MNPL	Micro- and nano-plastic
PET	Polyethylene terephthalate

## Appendix A

**Table A1:** Average total mass of polyethylene terephthalate plastics administered over 28 days for each dose group.

Average Total Plastic Consumption		
	Males (mg)	Females (mg)
Control	0.00	0.00
0.3 mg/kg/d	0.21	0.17
1.5 mg/kg/d	1.12	0.90
7.5 mg/kg/d	5.61	4.57
37.5 mg/kg/d	27.82	23.21

**Table A2:** Absolute organ weights in male mice exposed to PET-MPs.

			PET (mg/kg/d)		
	0	0.3	1.5	7.5	37.5
(g)					
Liver	1.434 ± 0.070	1.408 ± 0.038	1.437 ± 0.047	1.410 ± 0.048	1.369 ± 0.035
Kidneys	0.330 ± 0.007	0.333 ± 0.014	0.343 ± 0.012	0.328 ± 0.010	0.338 ± 0.010
Spleen	0.064 ± 0.002	0.066 ± 0.002	0.065 ± 0.002	0.066 ± 0.003	0.065 ± 0.002
Heart	0.137 ± 0.005	0.141 ± 0.004	0.138 ± 0.004	0.141 ± 0.006	0.136 ± 0.003
Lungs ^a^	0.225 ± 0.010	0.218 ± 0.012	0.226 ± 0.005	0.226 ± 0.012	0.223 ± 0.011
Seminal vesicles, coagulating glands, and prostate	0.417 ± 0.007	0.419 ± 0.007	0.439 ± 0.027	0.433 ± 0.017	0.433 ± 0.012
Stomach ^a^	0.213 ± 0.016	0.211 ± 0.008	0.218 ± 0.012	0.216 ± 0.011	0.205 ± 0.008
Small intestine ^a^	1.174 ± 0.030	1.130 ± 0.023	1.153 ± 0.028	1.121 ± 0.052	1.077 ± 0.024
Colon ^a^	0.224 ± 0.014	0.217 ± 0.011	0.220 ± 0.018	0.210 ± 0.008	0.214 ± 0.011
Brain ^a^	0.438 ± 0.005	0.438 ± 0.006	0.434 ± 0.005	0.432 ± 0.006	0.442 ± 0.005

Data are expressed as mean ± standard error of the mean for 10 mice/group with the following exceptions: lungs, stomach, small intestine, colon, and brain, *n* = 6 (^a^).

**Table A3:** Absolute organ weights in female mice exposed to PET-MPs.

			PET (mg/kg/d)		
	0	0.3	1.5	7.5	37.5
(g)					
Liver	1.079 ± 0.042	1.088 ± 0.034	1.018 ± 0.024	1.036 ± 0.028	1.099 ± 0.031
Kidneys	0.240 ± 0.007	0.238 ± 0.006	0.247 ± 0.011	0.229 ± 0.006	0.244 ± 0.007
Spleen	0.081 ± 0.004	0.077 ± 0.003	0.084 ± 0.005	0.079 ± 0.003	0.081 ± 0.004
Heart	0.116 ± 0.004	0.121 ± 0.003	0.119 ± 0.004	0.116 ± 0.002	0.118 ± 0.003
Lungs ^a^	0.204 ± 0.012	0.213 ± 0.010	0.199 ± 0.009	0.208 ± 0.005	0.187 ± 0.006
Uterus	0.113 ± 0.011	0.125 ± 0.006	0.107 ± 0.014	0.117 ± 0.012	0.124 ± 0.010
Stomach ^a^	0.200 ± 0.014	0.208 ± 0.006	0.219 ± 0.008	0.226 ± 0.005	0.210 ± 0.006
Small intestine ^a^	1.093 ± 0.047	1.144 ± 0.021	1.090 ± 0.027	1.108 ± 0.026	1.088 ± 0.031
Colon ^a^	0.235 ± 0.020	0.237 ± 0.010	0.225 ± 0.014	0.227 ± 0.019	0.234 ± 0.009
Brain ^a^	0.443 ± 0.003	0.445 ± 0.005	0.433 ± 0.003	0.434 ± 0.010	0.443 ± 0.007

Data are expressed as mean ± standard error of the mean for 10 mice/group with the following exceptions: lungs, stomach, small intestine, colon, and brain, *n* = 6 (^a^).
